# Built‐In Electric Field Accelerates Nanotopography‐Mediated Enhancement of Vascularized Osseointegration via Ca_v1.2_/Piezo/Ca^2+^/PI3K Signaling

**DOI:** 10.1002/smsc.202500095

**Published:** 2025-07-14

**Authors:** Jingyan Huang, Dongheng Lu, Cairong Xiao, Jiezhong Guan, Xiaoshuang Wang, Changhao Li, Peng Yu, Yan Wang

**Affiliations:** ^1^ Hospital of Stomatology Guanghua School of Stomatology Guangdong Provincial Key Laboratory of Stomatology Sun Yat‐sen University Guangzhou 510055 China; ^2^ School of Materials Science and Engineering, National Engineering Research Center for Tissue Restoration and Reconstruction South China University of Technology Guangzhou 510641 China

**Keywords:** angiogenesis, electric, mechanical, osseointegration, osteogenesis

## Abstract

Increasing studies have emphasized the role of implant surface modifications in enhancing osseointegration through the synergistic regulation of osteogenesis and angiogenesis. While both topography and electrical cues have been shown to promote these processes, the interplay between these biophysical characteristics and their combined effects remain unclear. This study employs polarized BaTiO_3_ nanorod arrays (NBTP) on titanium surfaces as model substrates to engineer a mechanobiological and piezoelectric microenvironment. Nanotopography improves hydrophilicity, piezoelectric properties, and surface potential due to sharp reduction in Young's modulus. In vitro experiments reveal that topography‐mediated mechanobiological remodeling primarily enhances osteogenesis in mesenchymal stem cells (MSCs) and angiogenesis in endothelial cells (ECs) via Piezo2/Piezo1/Ca^2+^ signaling. The augmented electric field further amplifies this mechanical stress‐driven osteogenic/angiogenic response by activating Ca_v1.2_ and potentiating Piezo2/Piezo1 signaling. Microarray analysis and blocking experiments identify the PI3K/AKT/mTOR/GSK3β pathway as a key mediator. Together, topography and the built‐in electric field activate paracrine crosstalk between MSCs and ECs, indirectly enhancing osteogenesis and angiogenesis. In vivo studies confirm that nanorod topography significantly improves vascularized osseointegration, while the built‐in electric field accelerates bone healing by remodeling the peri‐implant microenvironment. These findings advance the design of high‐performance bone implants by elucidating the mechanobiological–piezoelectric coupling mechanism underlying vascularized osteogenesis.

## Graphical abstract

Nanotopography improves the piezoelectric properties of BaTiO_3_ due to decreased Young's modulus. Nanotopography‐mediated mechanical stress dominantly boosts osteogenesis and angiogenesis via Piezo2/Piezo1/Ca^2+^ signaling. Augmented electric stimulation improves mechanical stress‐mediated osteogenic/angiogenic enhancement via PI3K/AKT/mTOR/GSK3β‐1 pathway. Topography and electric stimulation synergistically activate interaction between mesenchymal stem cells and endothelial cells. Electric stimulation accelerates nanotopography‐mediated vascularized osseointegration in rats.

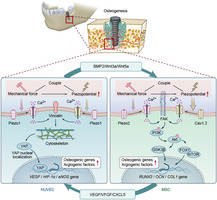

## 1 Introduction

Orthopedic implants are in high demand due to the increasing prevalence of bone diseases and tooth loss in aging populations. Rapid and stable long‐term osseointegration is essential for successful clinical outcomes and implant survival. This process relies on sufficient vascularized osteogenesis around the implant site. Consequently, the coupling of angiogenesis and osteogenesis in bone defect healing has gained significant attention in recent academic research. Increasing evidence indicates that in addition to transporting nutrients, oxygen, circulating cells, and waste products, blood vessels secrete factors that regulate bone regeneration and remodeling during the healing process.^[^ ^1^, ^2^ ^]^ Accordingly, considerable efforts have been made to modify implant surfaces to enhance angiogenesis–osteogenesis coupling. For example, Chen et al. demonstrated that pitavastatin‐loaded multilayer films on metal implants could activate the bone morphogenetic protein (BMP) and Wnt signaling pathways of human umbilical vein endothelial cells (HUVECs), stimulating paracrine secretion of angiogenic factors (Vascular Endothelial Growth Factor (VEGF), basic Fibroblast Growth Factor (bFGF), Platelet‐Derived Growth Factor (PDGF)‐bb, and Stromal Cell‐Derived Factor (SDF)‐1α) from mesenchymal stem cells (MSCs). Through this paracrine crosstalk, the films indirectly increased the angiogenic potential of MSCs and the osteogenic potential of endothelial cells (ECs).^[^ ^1^ ^]^

High‐fidelity replication of the biophysical microenvironment of natural bone extracellular matrices (ECMs) represents an effective approach for the rational design of bone implants. Current advancements primarily focus on simulating individual biophysical cues, such as topology or electrical properties, which enhance osseointegration to some degree. However, achieving rapid osteogenic performance remains challenging. The bone ECM exhibits moderate mechanical properties from its intrinsic nanotopography and unique electrical characteristics conferred by piezoelectric collagen fibers. Increasing evidence highlights the crucial roles of both topography and electrical signaling in bone regeneration. Surface morphology, particularly nanotopography, stimulates cell cytoskeleton and focal adhesion remodeling.^[^ ^3^, ^4^ ^]^ The resulting cytoskeletal tension promotes mechanotransduction, translating mechanical stimuli into biochemical signals that activate multiple intracellular pathways, such as β1‐integrin binding/clustering, myosin–actin interactions, and signaling through Rho‐associated protein kinase (ROCK) and Mitogen‐Activated Protein Kinase (MEK)–Extracellular Signal‐Regulated Kinase (ERK) pathways,^[^ ^3^ ^]^ ultimately accelerating osteogenic differentiation. In addition, the endogenous electric fields transmit electrical signals to bone marrow mesenchymal stem cells (BMSCs), influencing osteogenic differentiation through the activation of Piezo1 proteins and subsequent calcium,^[^ ^5^ ^]^ the Kyoto Encyclopedia of Genes and Genomes (KEGG),^[^ ^6^ ^]^ and Yes‐Associated Protein (YAP) signaling pathways.^[^ ^7^ ^]^ Numerous studies have confirmed the modulatory effects of electrical stimulation on bone regeneration.^[^ ^8^, ^9^, ^10^ ^]^ For instance, Cai et al. reported that a piezoelectric barium titanate (BT) coating activated by external pulsed ultrasound improved MC3T3‐E1 cell activity by opening L‐type calcium channels and increasing intracellular calcium.^[^ ^11^ ^]^ However, reliance on external excitation devices limits the practical application of piezoelectric biomaterials in terms of convenience and safety.

Although implant engineering incorporating both surface topography and electrical activity has been extensively studied, research on the orchestration of spatial and electrical cues for high‐fidelity simulation of the microenvironment—particularly for myogenic precursor cells,^[^ ^12^ ^]^ neural stem cells,^[^ ^13^ ^]^ and B MSCs^[^ ^14^ ^]^—remains ongoing. Current studies typically treat topography and electric cues as independent factors, leaving their interactions poorly understood. Critical questions remain unanswered: Which cues dominate osteogenesis? How do spatiotemporal topography–electrical interactions regulate angiogenesis–osteogenesis coupling?

Given that topography represents a more stable and inherent biophysical cue,^[^ ^15^ ^]^ we hypothesized that nanotopography plays a dominant role in vascularized osteogenesis, while the built‐in electric field accelerates this process. To verify this assumption, we employed piezoelectric BaTiO_3_ nanorod arrays as a model substrate to engineer a combined mechanobiological and built‐in electrical microenvironment. We constructed a BaTiO_3_ nanorod array coating on titanium implants and thoroughly characterized its surface and mechanical–physical characteristics. The osteogenic differentiation capacity of MSCs and the angiogenic potential of HUVECs were then evaluated under these conditions. To elucidate the underlying mechanisms, we performed microarray analysis, conducted blocking experiments, and assessed mechanical‐related factors in the mechanobiological and piezoelectric microenvironment. We further investigated the paracrine crosstalk between MSCs and HUVECs under these engineered conditions. Finally, we validated our findings through chorioallantoic membrane (CAM) assays and femur implantation studies to evaluate angiogenesis and osteogenesis surrounding the polarized BaTiO_3_ nanorod‐coated titanium implants in vivo. This comprehensive research provides valuable insights for developing next‐generation, high‐performance bone implants.

## 2 Results and Discussion

### 2.1 The Characterization of Polarized BaTiO_3_ Nanorod Arrays

**Figure 1:**
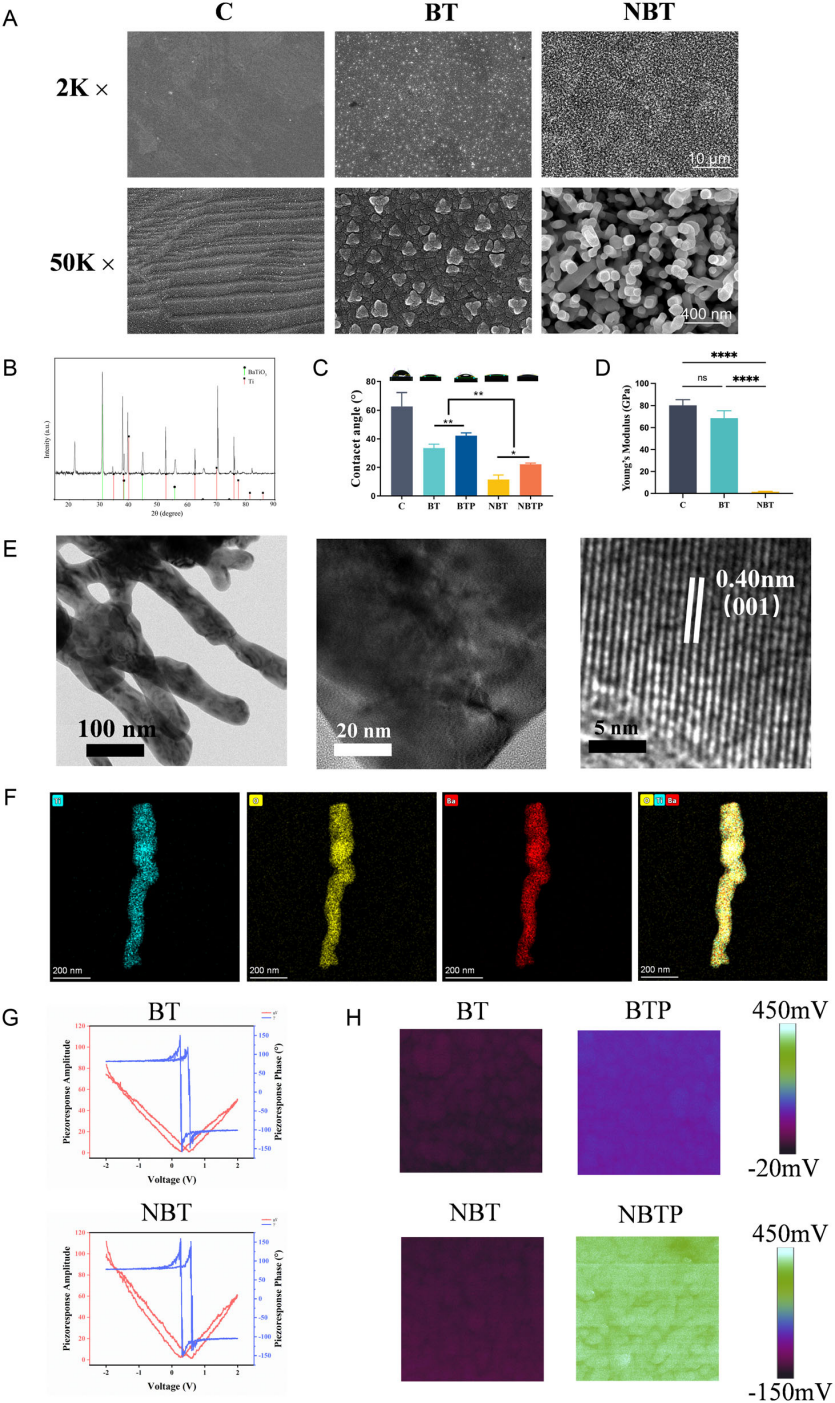
Characterization of polarized BaTiO_3_ nanorod arrays. A) SEM surface morphology of C, BT, and NBT samples. B) XRD pattern of NBTP surface. C) Water contact angle measurements for C, BT, polarized barium titanate nanorod arrays (BTP), NBT, and NBTP. D) Young's modulus of C, BT, and NBT. E) High‐resolution TEM image showing NBT nanorod array microstructure. F) TEM–EDS element mapping of NBT nanorod. G) The corresponding piezoresponse force microscopy phase (blue line) and amplitude (red line) hysteresis loops for the BT and NBT. H) Surface potential measurements of BTP and NBTP.

BaTiO_3_ is a well‐known piezoelectric ceramic material that has gained significant attention in biomedical applications due to its remarkable piezoelectric properties and acceptable biocompatibility. Its piezoelectric effect arises from the noncentrosymmetric crystal structure, which generates electric charge under mechanical stress.^[^ ^16^ ^]^ To precisely analyze the surface morphology of BaTiO_3_ nanorod arrays integrated onto the titanium substrate, scanning electron microscopy (SEM) was employed. Due to the presence of a rich oxide layer on the pure Ti surface, it exhibited a relatively smooth morphology under low‐magnification SEM (**Figure** **1**A). The wavy striations observed on the pure titanium (C) surface through high‐magnification SEM may be attributed to mechanical processing techniques such as grinding, polishing, or machining. Regular nanometer‐sized particles are visible on the BT surface, which may be related to the cubic‐tetragonal transformation of BT.^[^ ^17^, ^18^ ^]^ The NBT surface exhibited orderly oriented BaTiO_3_ nanorods, indicating uniform distribution across the Ti surface. X‐ray powder diffraction (XRD) analysis revealed characteristic peaks of BaTiO_3_, confirming the successful construction of BaTiO_3_ nanorod arrays through elemental analysis of the material surface (Figure 1B). The average water contact angle of Ti was nearly twice that of BT and four times of NBT, indicating that BaTiO_3_ enhances the hydrophilicity of the material surface, with BaTiO_3_ nanorod arrays maximizing this effect. However, the hydrophilicity of both polarized BT and NBT decreased to varying degrees, which was associated with the increased surface potential after polarization (Figure 1C). HRTEM images revealed the d‐spacing of perovskite BaTiO_3_ crystal planes with lattice fringes of 0.4023 nm (Figure 1E). The transmission electron microscopy (TEM)–Energy Dispersive X‐ray Spectroscopy (EDS) analysis of a single nanorod revealed that the BaTiO_3_ nanorods in NBT are ≈500 nm in height, with Ba, Ti, and O elements uniformly distributed throughout the nanorod (Figure 1F).

Moreover, the nanoscale morphology of a Ti implant surface is strongly correlated with its mechanical properties. Young's modulus of NBT (1.46 ± 0.22 GPa) was significantly lower than that of C (80.25 ± 3.00 GPa) and BT (68.61 ± 3.9 GPa), closely matching Young's modulus of human bone (1–22.3 GPa), according to the nanoindentation test. This gives NBT enhanced biocompatibility as a potential replacement material^[^ ^19^ ^]^ (Figure 1D). The Young's modulus of a material influences its piezoelectric performance by affecting its deformation under mechanical stress. Materials with lower Young's modulus deform more easily, enhancing electric charge generation under stress and improving electrical properties.^[^ ^20^ ^]^ We therefore utilized piezoresponse force microscopy (PFM) to characterize the piezoelectric performance of NBT and BT. Cantilever oscillations were recorded as phase and amplitude signals. A 180° phase switching and hysteresis loop local polarization‐electric field (P–E) loops of NBT and BT confirmed their ferroelectricity, providing theoretical support for polarization under applied electric fields (Figure 1G). The butterfly‐shaped curve showed that NBT exhibited significantly greater deformation than BT under identical voltage stimulation, demonstrating superior piezoelectric properties (Figure 1G)—consistent with Young's modulus results. Under external electric fields, dipole moments in the noncentrosymmetric ferroelectric crystals reorient, forming ferroelectric hysteresis loops. Ultimately, all dipoles align along the polarization direction, generating piezoelectric potential.^[^ ^21^, ^22^ ^]^ Scanning Kelvin probe microscopy revealed that after a 0.4 kV polarization, NBTP and polarized barium titanate (BTP) showed significantly higher surface potentials than NBT and BT. In contrast to BTP (130 mV), the surface potential of NBTP (373 mV) increased significantly, further indicating that NBT has superior ferroelectric properties (Figure 1H). These data suggest that the NBTP nanorod arrays can generate high‐intensity electrical signals while providing nanoscale topographical stimulation, creating an orchestrated mechanobiological and piezoelectric microenvironment conducive to tissue regeneration.

### 2.2 Built‐In Electric Field Improves Mechanical‐Stress‐Mediated Enhancement of Osteogenic Potential of MSCs

**Figure 2:**
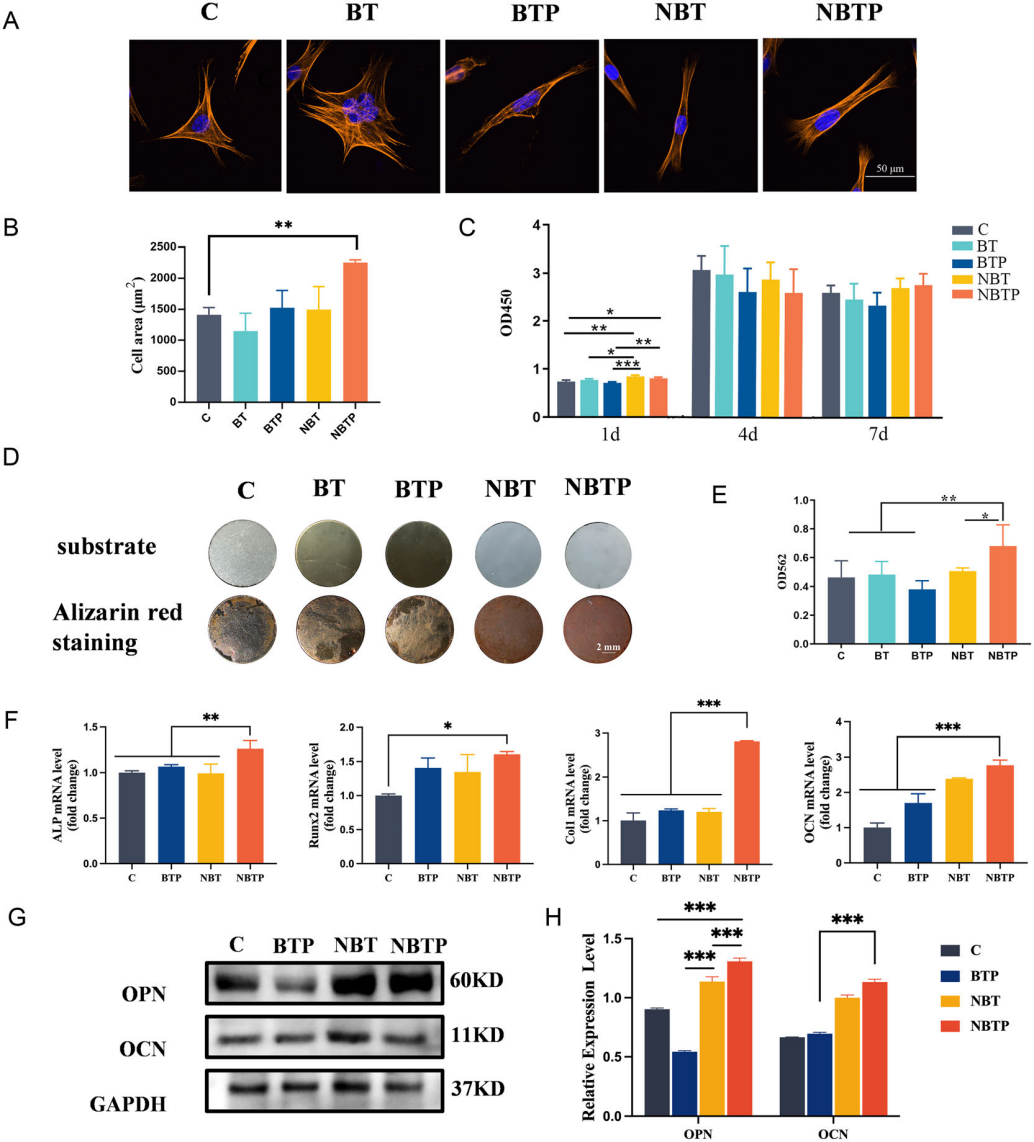
NBT and NBTP surfaces promote BMSC elongation, proliferation, and ECM mineralization. A) Confocal fluorescence images of the cytoskeleton (orange) and nucleus (blue) of BMSCs cultured for 24 h on different surfaces. B) Corresponding semiquantitative analysis of the cell spreading area. C) BMSC proliferation on each surface after 1, 4, and 7 days of culture. D) Alizarin Red S (ARS) staining of BMSCs cultured on different surfaces for 14 days. E) Semiquantitative analysis of ARS staining. F) mRNA expression of osteogenic genes (Alkaline Phosphatase (ALP), Runx2, Col1, Osteocalcin (OCN)). G) Western blot analysis of OPN, OCN, and Glyceraldehyde 3‐Phosphate Dehydrogenase (GAPDH) protein expression. H) Quantitative analysis of OPN/GAPDH and OCN/GAPDH ratios. ANOVA followed by Tukey's post hoc test was performed for statistical analysis (error bar: ±SD; *n* = 3; *p < 0.05, **p < 0.01, ***p < 0.001).

Cytoskeletal staining demonstrated favorable BMSC spreading on NBTP, indicating more elongated cellular morphology compared to other groups. While BMSCs exhibited triangular/polygonal morphology on C and BT, they showed elongated shapes on BTP and spindle‐like morphology on NBT and NBTP (**Figure** **2**A). Statistical analysis revealed significantly higher adhesive areas for cells on NBT and NBTP (2239.16 μm^2^) versus other groups, indicating enhanced cellular expansion (Figure 2B). These morphological alterations can be attributed to cytoskeletal reorganization through actin and microtubule remodeling,^[^ ^23^ ^]^ induced by the nanorod surface topography. The resulting tensional stimuli enhanced micromechanical forces, promoting directed cytoplasmic boundary translation^[^ ^24^ ^]^ that ultimately produced the observed spindle‐shaped morphology. These findings indicate that the nanorod cue directs MSC morphology and can be converted into biomechanical cues.

Biocompatibility is a fundamental requirement for implant material. In proliferation assays at days 4 and 7, no significant differences were observed between materials, indicating comparable cell viability on NBTP and NBT versus Ti, BT, and BTP (Figure 2C). These results confirm adequate biocompatibility across all test materials.

To understand the electromechanobiological microenvironment created by NBTP for osteogenic differentiation, we assessed BMSC osteogenic capacity through the comprehensive analysis of ECM mineralization and osteogenic gene expression. Alizarin Red S, an anthraquinone‐derived dye, is commonly used to detect calcium deposits in mineralized tissues. Calcium nodules, key indicators of late‐stage osteogenic differentiation, bind to Alizarin Red S through chelation. This reaction forms red Alizarin–calcium complexes—birefringent compounds that appear as red deposits under optical microscopy.^[^ ^25^ ^]^ Alizarin Red S staining revealed significant differences in mineralization across materials. NBTP‐cultured cells demonstrated robust calcium accumulation, indicating enhanced mineralization, while Ti group exhibited minimal deposition (Figure 2D). Quantification confirmed that NBTP significantly increased calcium nodule formation (*P *< 0.05), demonstrating its superior osteogenic potential through combined mechanical and electrical signals (Figure 2E).

To investigate why NBTP promotes ECM mineralization, we measured key osteogenic genes/proteins using PCR and western blotting. Alkaline Phosphatase (ALP) is an early marker of osteoblast differentiation. Osteocalcin (OCN), a noncollagenous protein produced by osteoblasts, regulates mineralization and bone matrix maturation. Its expression indicates mature osteoblast function and serves as a reliable osteogenesis marker.^[^ ^26^ ^]^ Col1, the most abundant collagen family protein, is a fundamental ECM component.^[^ ^27^ ^]^ In osteogenesis, Col1 maintains skeletal structural integrity and mechanical strength providing a scaffold for mineral deposition and bone formation and remodeling. Its triple‐helical structure confers bone tissues tensile strength, enhancing resilience against mechanical stress.^[^ ^28^ ^]^ The coordinated expression of OCN, osteopontin (OPN), and Col1 marks BMSC osteogenic progression, revealing molecular events underlying bone formation.^[^ ^29^ ^]^ Expression of ALP, Runx2, Col1, OCN, and OPN genes/proteins was elevated in NBTP‐cultured BMSCs (*P *< 0.05; Figure 2F–H). BTP showed a slightly increased expression of ALP, RUNX2, Col1, and OCN (Figure 2F). For NBT, RUNX2 and OCN gene expression (Figure 2F) and OPN and OCN protein expression (Figure 2G,H) were promoted, despite lacking statistical significance. This suggested that electrical cues positively influence early osteogenic differentiation, while nanotopographic cues dominantly promote later stages. The electric‐nanotopography combination amplifies electric signals, benefiting both differentiation stages. Overall, topography‐mediated mechanobiological remodeling predominantly enhances MSC osteogenic potential compared to electrical cues alone. Nanotopography‐augmented electric activity further improves mechanical‐stress‐mediated osteogenic enhancement.

### 2.3 Electro‐Mechanobiological Coupling Increases Intracellular Ca^2+^ Concentration in BMSCs via Ca_v1.2_ and Piezo2 Activation

**Figure 3:**
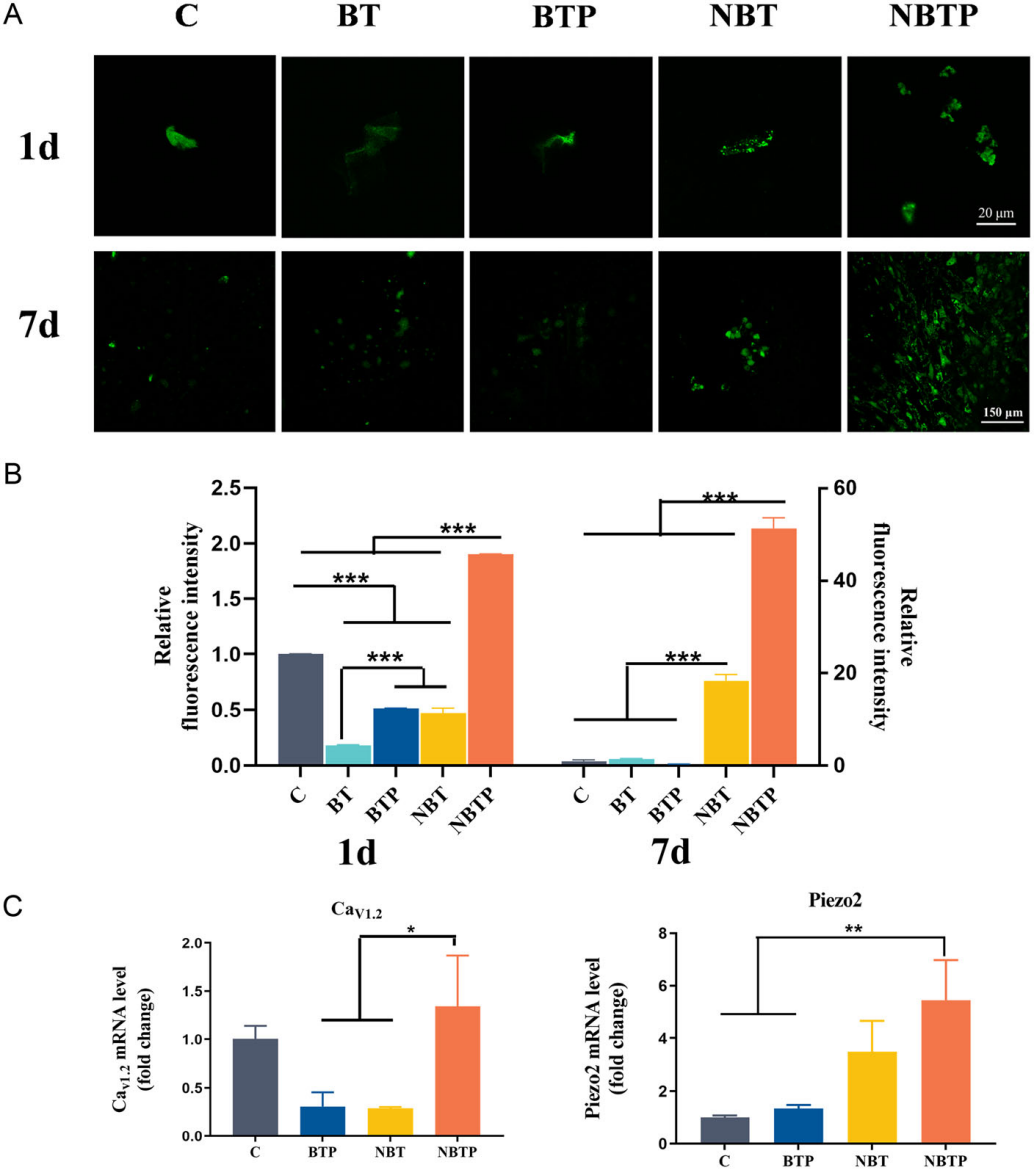
NBTP increases intracellular Ca^2+^ concentration in BMSCs through Cav_1.2_ /Piezo2 channel activation. A) Fluo‐4 AM fluorescence showing calcium concentration in BMSCs. B) Semiquantitative analysis of intracellular calcium levels. C) mRNA expression of Ca_v1.2_ and Piezo2 in BMSCs cultured on different surfaces. ANOVA followed by Tukey's post hoc test was performed for statistical analysis (error bar: ±SD; *n* = 3; *p < 0.05, **p < 0.01, ***p < 0.001).

To visualize intracellular Ca^2+^ concentration variations across material groups, we used Fluo‐4 AM fluorescent probe. On day 1, processed BT coatings elevated Ca^2+^ concentrations compared to plain BT coatings, demonstrating that both nanotopography‐induced mechanical forces and electrical signals stimulate calcium fluxes (**Figure** **3**A,B). By day 7, NBTP and BTP maintained significantly higher intracellular Ca^2+^ concentrations than BT and C groups. Notably, NBTP showed the highest Ca^2+^ concentrations at both time points (*p *< 0.001), demonstrating the synergistic effect of combined mechanical and electrical signals on Ca^2+^ elevation.

To investigate the calcium flux activation pathways, we measured relevant ion channel expression. Results showed a significant upregulation Ca_v1.2_ and Piezo2 mRNA in NBTP‐cultured cells versus other groups (*p *< 0.05; Figure 3C). NBT specifically elevated Piezo2 expression, while BTP showed no significant changes in either channel. Ca_v1.2_, voltage‐dependent L‐type calcium channel subunit, critically regulates calcium influx by modulating membrane potential.^[^ ^30^ ^]^ Piezo2, a mechanosensitive channel activated by mechanical stimuli, is essential for mechanotransduction in mammals.^[^ ^31^ ^]^ Its structural components facilitate channel opening under mechanical force. The elevated Ca_v1.2_ expression observed with NBTP suggests that electrical activity from polarized nanotopography activates calcium channels (Figure 3C). Ca_v1.2_ remained inactive on BTP, probably due to insufficient surface potential. Meanwhile, the increased Piezo2 expression on NBTP and NBT (*P *< 0.05) reflects nanorod‐induced micromechanical forces (Figure 3C). NBTP further enhanced Piezo2 activation compared to NBT, demonstrating that electric stimulation augments the effects mediated by nanotopography. Therefore, nanotopography‐augmented electrical stimulation synergistically activates Piezo2 while triggering Ca_v1.2,_ collectively promoting calcium influx.

### 2.4 The PI3K–AKT Signaling Axis is Upregulated in BMSCs within an Orchestrated Mechanobiological and Piezoelectric Microenvironment

Having established that the coordinated mechanobiological–piezoelectric microenvironment regulates osteogenic differentiation, we investigated how polarized BaTiO_3_ nanorod arrays influence this process. Gene microarray analysis was performed to analyze temporal mRNA expression patterns in BMSC cultured on different substrates. Principal component analysis (PCA) demonstrated satisfactory intragroup reproducibility for the C, BT, BTP, NBT, and NBTP groups (Figure S1, Supporting Information). Of note, BT and BTP samples showed cross‐distribution, while clear separation was observed among C, NBT, and NBTP clusters. These results indicated similarity between BT and BTP. Accordingly, BT was excluded and BTP was selected as a control for subsequent experiments.

**Figure 4:**
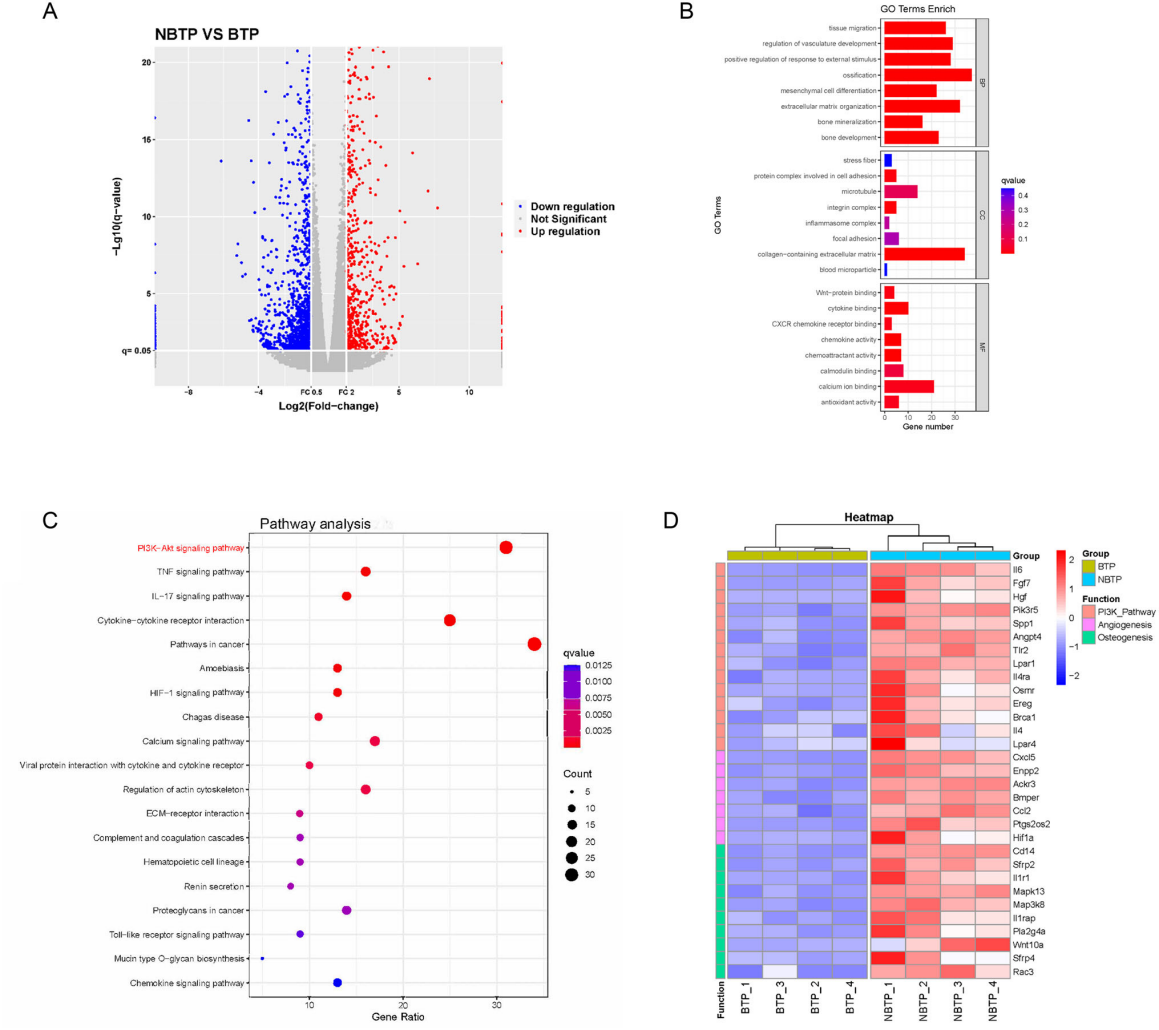
Transcriptome analysis of MSCs. A) Quantitative analysis of differentially expressed genes (DEGs) in MSCs from the BTP and NBTP groups. B) GO enrichment analysis of the up‐ and downregulated biological processes, cellular component, and molecular function. C) KEGG pathway analysis of MSCs from the BTP and NBTP groups. D) Heatmap of DEGs associated with immune regulation.

Next, we analyzed the differential gene expression between the NBTP and BTP groups. Comparison revealed 631 differentially expressed genes (324 upregulated, 307 downregulated) between NBTP and BTP (**Figure** **4**A).

The Gene Ontology (GO) database is a widely used bioinformatic system that encompasses three hierarchical aspects of biology: cellular components, molecular functions, and biological processes (Figure 4B). In the NBTP group, biological processes related to bone development—including ossification, mesenchymal cell differentiation, and bone mineralization—showed significant enrichment. Processes regulating vascular development were also elevated (Figure 4B). The ECM, primarily composed of collagen proteins and glycosaminoglycans, serves as a vital physical scaffold and provides essential biochemical and biomechanical cues for tissue morphogenesis, differentiation, and homeostasis.^[^ ^32^ ^]^ The upregulated enrichment of ECM organization underscores the influence of NBTP nanotopography compared to that of BTP (Figure 4B). In the cellular component category, upregulated subcategories included collagen‐containing ECM, microtubules, and integrin complexes—all closely related to mechanotransduction. Molecular function analysis showed increased enrichment in calcium ion binding and calmodulin binding, validating calcium channel activation mechanisms consistent with our previous results (Figure 4B). GO analysis suggests that NBTP influences collagen‐containing ECM through combined mechanobiological and piezoelectric effects on nanomorphology and electrical signaling, thereby promoting osteogenesis.

Based on gene function, we classified the differentially expressed gene into three categories: PI3K pathway‐related, osteogenesis‐related, and angiogenesis‐related (Figure 4D). The osteogenesis‐related genes included Cd14, Sfrp2, Mapk13, Map3k8, Wnt10a, IL1r1, IL1rap, Cxcl1, Pla2g4a, and Rac3 (Figure 4D). Wnt10a, a member of the WNT gene family, plays an essential role in osteoblast differentiation and bone metabolism.^[^ ^33^ ^]^ As a key activator of Wnt/β‐catenin signaling, Wnt10a enhances osteogenic differentiation and regulate cell proliferation and migration by stabilizing the downstream β‐catenin pathway.^[^ ^34^, ^35^, ^36^ ^]^ In contrast, BMSCs cultured on NBTP also upregulated angiogenesis‐related genes such as CXCL5, CCL2, and ACKR3. CXCL5, also known as epithelial‐derived neutrophil‐activating peptide 78 (ENA‐78), is a member of the CXC chemokine family.^[^ ^37^ ^]^ It is primarily secreted by epithelial cells, functions as a neutrophil chemoattractant, and plays roles in inflammation, angiogenesis, wound healing, and immune responses.^[^ ^38^ ^]^ In bone biology, CXCL5 contributes to osteogenesis and remodeling.^[^ ^39^, ^40^ ^]^ Xu et al. demonstrated that mesenchymal stromal cells can promote CXCL5 expression via CCL7 secretion, thereby enhancing angiogenesis and vascular metastasis.^[^ ^41^ ^]^ CCL2, also known as monocyte chemoattractant protein‐1 (MCP‐1), recruits monocytes, memory T cells, and dendritic cells to sites of injury or inflammation.^[^ ^40^ ^]^ Stamatovic et al. showed that CCL2 induces angiogenesis by upregulating Ets‐1 mRNA expression and enhancing DNA‐binding activity.^[^ ^42^ ^]^ Additionally, PI3K pathway‐related genes (e.g., Fgf7, Spp1, Angpt4) were upregulated.^[^ ^43^ ^]^ Together, these finding suggest that the mechanobiological and piezoelectric microenvironment orchestrates immune cell recruitment, immune response modulation, and angiogenesis to promote osseointegration.

**Figure 5:**
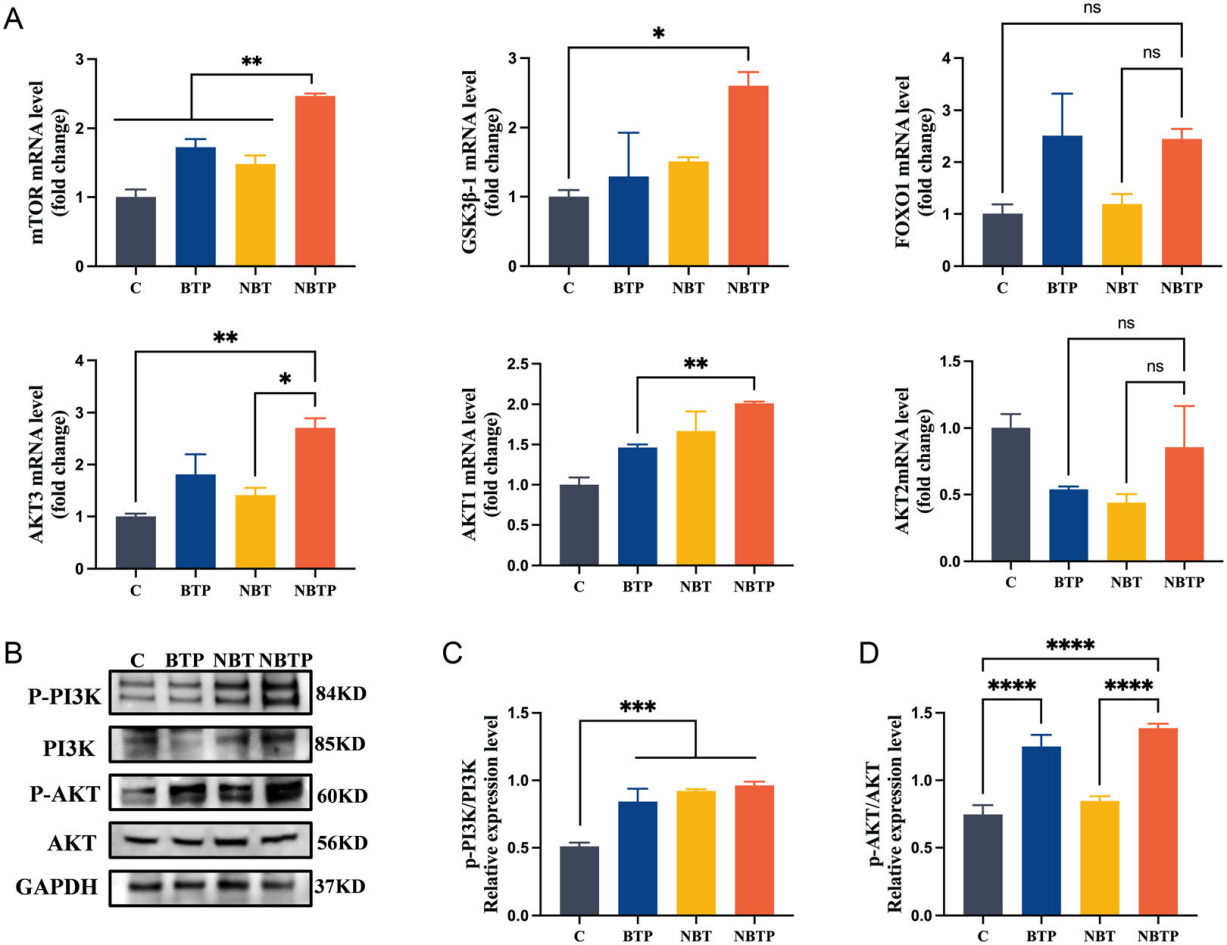
mRNA and protein expression profiles of osteogenesis‐related pathway. A) mRNA expression of osteogenic factors (AKT1, AKT2, AKT3, mTOR, GSK3β, and FOXO1) in BMSCs cultured on each surface. B) Western blotting analysis of *p*‐PI3K, PI3K, *p*‐AKT, AKT, and GAPDH protein expression. C) Quantitative analysis of *p*‐PI3K/PI3K ratios. D) Quantitative analysis of *p*‐AKT/AKT ratios. ANOVA followed by Tukey's post hoc test was performed for statistical analysis (error bar: ±SD; *n* = 3; *p < 0.05, **p < 0.01, ***p < 0.001, ****p < 0.0001).

Subsequently, pathway enrichment analysis indicated that the PI3K–AKT pathway correlated with the upregulated osteogenic marker expression (Figure 4C). The PI3K–AKT pathway, a classical osteogenic pathway, regulates cellular processes such as proliferation, survival, differentiation, and metabolism.^[^ ^44^ ^]^ Its activation modulates key molecular mechanisms in bone formation and maintenance.^[^ ^45^, ^46^, ^47^, ^48^ ^]^ In this study, PI3K protein expression was significantly upregulated in the NBTP group (**Figure** **5**B). Similarly, NBTP promoted mRNA expression of AKT1, KT3, and downstream genes (mTOR, GSK3β; Figure 5A). While PI3K–AKT pathway activation was observed in BTP and NBT groups, it was less pronounced, underscoring the augmented osteogenic potential of the NBTP group versus controls. Phosphorylation is crucial for PI3K/AKT activation. In this pathway, AKT phosphorylation at specific residues regulates cellular growth, survival, and metabolism,^[^ ^48^ ^]^ transmitting extracellular signals to drive proliferation and differentiation.^[^ ^49^ ^]^ Notably, p‐PI3K/PI3K ratios in BMSCs from the BTP, NBT, and NBTP groups were significantly higher than in the C group (*p *< 0.001) (Figure 5B&C). p‐AKT/AKT levels were also significantly higher in NBTP than in the NBT and control groups (*p *< 0.001) (Figure 5D), confirming pathway activation via phosphorylation. These findings align with prior studies. Chen et al. reported that PI3K/AKT inhibition (via LY294002) suppressed Wnt/β‐catenin by reducing GSK3β phosphorylation, impairing BMSC osteogenic differentiation.^[^ ^50^ ^]^ Similarly, Zhao et al. and Hu et al. underscored the critical role of the PI3K/AKT/GSK3β signaling pathway in facilitating BMSC osteogenic differentiation.^[^ ^48^, ^51^ ^]^

In contrast, mTOR (particularly mTORC1) regulates bone homeostasis by balancing formation and resorption.^[^ ^52^ ^]^ Activated by PI3K/AKT and anabolic Wnt ligands (e.g., Wnt3a, Wnt7b), mTORC1 promotes osteoblast differentiation and bone formation. Its inhibition may cause trabecular bone loss,^[^ ^53^ ^]^ while its role in osteoclasts—although complex and under active investigation—further underscores its nuanced impact on bone metabolism.^[^ ^54^ ^]^

In our study, we found that NBTP established a mechanobiological–piezoelectric microenvironment that facilitated calcium ion influx, opening of Ca_v1.2_ and Piezo2 channels, osteogenic differentiation, and PI3K/AKT signaling activation. Within this specialized microenvironment, calcium ions (Ca^2+^) served as pivotal messengers for transmitting electrical and biomechanical signals. Bhattarai et al. developed an electrically conductive polyaniline substrate coated with titanium oxide nanotubes, which significantly improved preosteoblast attachment, proliferation, and differentiation.^[^ ^55^ ^]^ Similarly, Huang et al. fabricated titanium dioxide nanorod/bismuth oxide nanodot heterojunctions on bone implant surfaces. By monitoring intracellular calcium concentration, PI3K expression, and YAP intracellular localization, they demonstrated that nanoheterojunction‐derived electrical and micromechanical cues are transduced into cellular responses via the PI3K pathway, ultimately upregulating osteogenesis‐related genes.^[^ ^14^ ^]^

### 2.5 Calcium Channel Blockade Eliminated the Superiority of NBTP on Intracellular Calcium Levels, PI3K/AKT Pathway Expression, and Osteogenic Differentiation of MSCs

**Figure 6:**
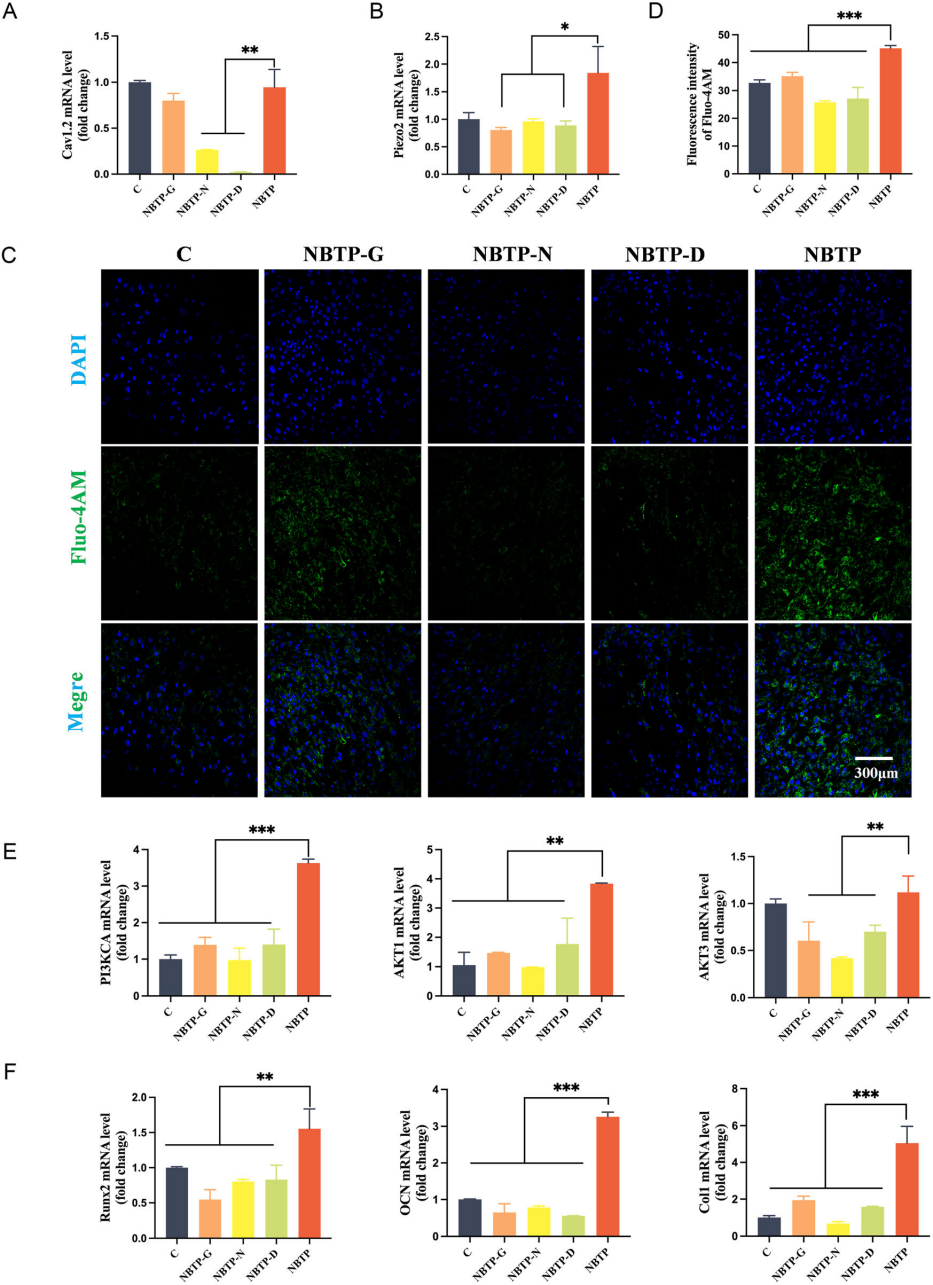
Calcium channel blockade by GsMTx4/nifedipine eliminates the enhanced effects of NBTP on intracellular calcium levels, PI3K/AKT pathway expression, and osteogenic differentiation of MSCs. A) mRNA expression of Ca_v1.2_ in BMSCs cultured on each surface following treatment with GsMTx4/nifedipine. B) mRNA expression of Piezo2 in BMSCs under treatment conditions. C) Confocal fluorescence images showing intracellular calcium levels in BMSCs after treatment. D) Semiquantitative analysis of intracellular calcium fluorescence intensity. E) mRNA expression PI3K–AKT pathway‐related genes (PI3KCA, AKT1, AKT3). F) mRNA expression of osteogenesis‐related genes (Runx2, Col1, OCN). ANOVA followed by Tukey's post hoc test was performed for statistical analysis (error bar: ±SD; *n* = 3; *p < 0.05, **p < 0.01, ***p < 0.001).

To elucidate the causal relationship between these biological processes, we performed blocking experiments by adding the Ca_v1.2_ inhibitor nifedipine and the Piezo2 antagonist GsMTx4 to the medium in the NBTP group separately and in combination.^[^ ^56^, ^57^ ^]^ As expected, quantitative real‐time polymerase chain reaction (RT‐qPCR) results showed that Ca_v1.2_ expression on the NBTP group decreased significantly by 50% or even 100% after treatment with nifedipine alone or the nifedipine/GsMTx4 combination (*P *< 0.01; **Figure** **6**A). Similarly, Piezo2 expression on NBTP was significantly reduced by 50% following treatment with GsMTx4 alone or in combination (*P *< 0.05; Figure 6B). These results confirmed that nifedipine and GsMTx4 could effectively inhibit Ca_v1.2_ and Piezo2 expression, respectively. We next examined the effect of blocking Ca_v__1.2_ and Piezo2 channels on Ca^2+^ concentration, osteogenic differentiation, and PI3K signaling. Notably, Ca^2+^ concentration decreased significantly after adding the channel blockers, with the most pronounced reduction observed with nifedipine and the combination treatment (*P *< 0.05; Figure 6C,D). This suggests that electrically induced activation of Ca_v1.2_ plays a dominant role in the Ca^2+^ influx upregulation, while nanotopography‐induced activation of Piezo2 provides synergistic modulation in the complex mechanobiological–piezoelectric microenvironment. Furthermore, expression levels of AKT1, AKT3, and the PI3K pathway subunit gene PI3KCA in NBTP were significantly reduced to levels comparable to Group C following treatment with either blocker alone or in combination (*P *< 0.05; Figure 6E). A similar trend was observed for osteogenesis‐related genes (Runx2, OCN, and Col1) after blocker treatment (*P *< 0.05; Figure 6F). Based on these observations, we conclude that in the orchestrated mechanobiological and piezoelectric microenvironment, Ca_v__1.2_ dominantly upregulates Ca^2+^ influx, which activates PI3K/AKT/mTOR/GSK3β and osteogenic‐related signaling pathways. In this context, Piezo2 exerts a synergistic effect.

### 2.6 The Built‐In Electric Field Enhances Mechanical‐Stress‐Mediated Angiogenesis of HUVECs via Piezo1/Ca^2+^ Signaling

We first examined cell morphology by staining nuclei and F‐actin cytoskeletons of HUVECs cultured on different surfaces. HUVECs on NBT and NBTP surfaces exhibited more rounded morphologies (aspect ratio closer to 1), while those on Ti and BTP displayed irregular spindle shapes (**Figure** **7**A,B). The smaller nucleus‐to‐cytoplasm ratio and larger cell area observed on NBT and NBTP surfaces suggested greater adhesion and spreading compared to those on Ti and BTP (Figure 7C). Cells Count Kit‐8 (CCK8) assays revealed comparable proliferation rates across all groups after 1, 4, and 7 days of culture, confirming the biocompatibility of all Ti substrates (Figure 7D). In tube formation assays, HUVECs on NBT and NBTP formed more intricate and cohesive vascular network than those on Ti and BTP, with NBTP particularly robust network formation characterized by more nodes, junctions, branches, and longer tubes (Figure 7E,F). Gene expression analysis showed significantly higher levels of eNOS and VEGF mRNA in HUVECs on NBTP compared to other groups, with BTP and NBT showing intermediate expression. Hypoxia‐Inducible Factor (HIF)‐1α expression was elevated in NBT and NBTP groups compared to that in Ti and BTP (Figure 7G). Immunofluorescence staining confirmed these findings at the protein level, with NBTP and NBT showing stronger VEGF, eNOS, and CD31 than Ti and BTP (Figure 7H). These results demonstrate that the built‐in electric field improves mechanical‐stress‐mediated angiogenesis in HUVECs.

Figure 7NBT and NBTP surfaces promote HUVEC spreading, proliferation, and angiogenesis. A) Confocal fluorescence images of HUVEC cytoskeleton (F‐actin, yellow) and nuclei (blue) after 24 h of culture on different surfaces. B) Semiquantitative analysis of cell aspect ratio. C) Semiquantitative analysis of nucleus‐to‐cytoplasm ratio. D) CCK‐8 proliferation assay on each surface after seeding for 1, 4, and 7 days. E) Tube formation assay after 7 days of culture. F) Semiquantitative analysis of tube formation parameters. G) mRNA expression of angiogenesis‐related genes (eNOS, VEGF, HIF‐1α). H) Immunofluorescence staining of CD31, VEGF, and eNOS and corresponding semiquantitative analysis. I) Immunofluorescent staining of Piezo1, vinculin, YAP, and Ca^2+^ and corresponding semiquantitative analysis. ANOVA followed by Tukey's post hoc test was performed for statistical analysis (error bar: ±SD; *n* = 3; *p < 0.05, **p < 0.01, ***p < 0.001, ****p < 0.0001).

**Figure:**
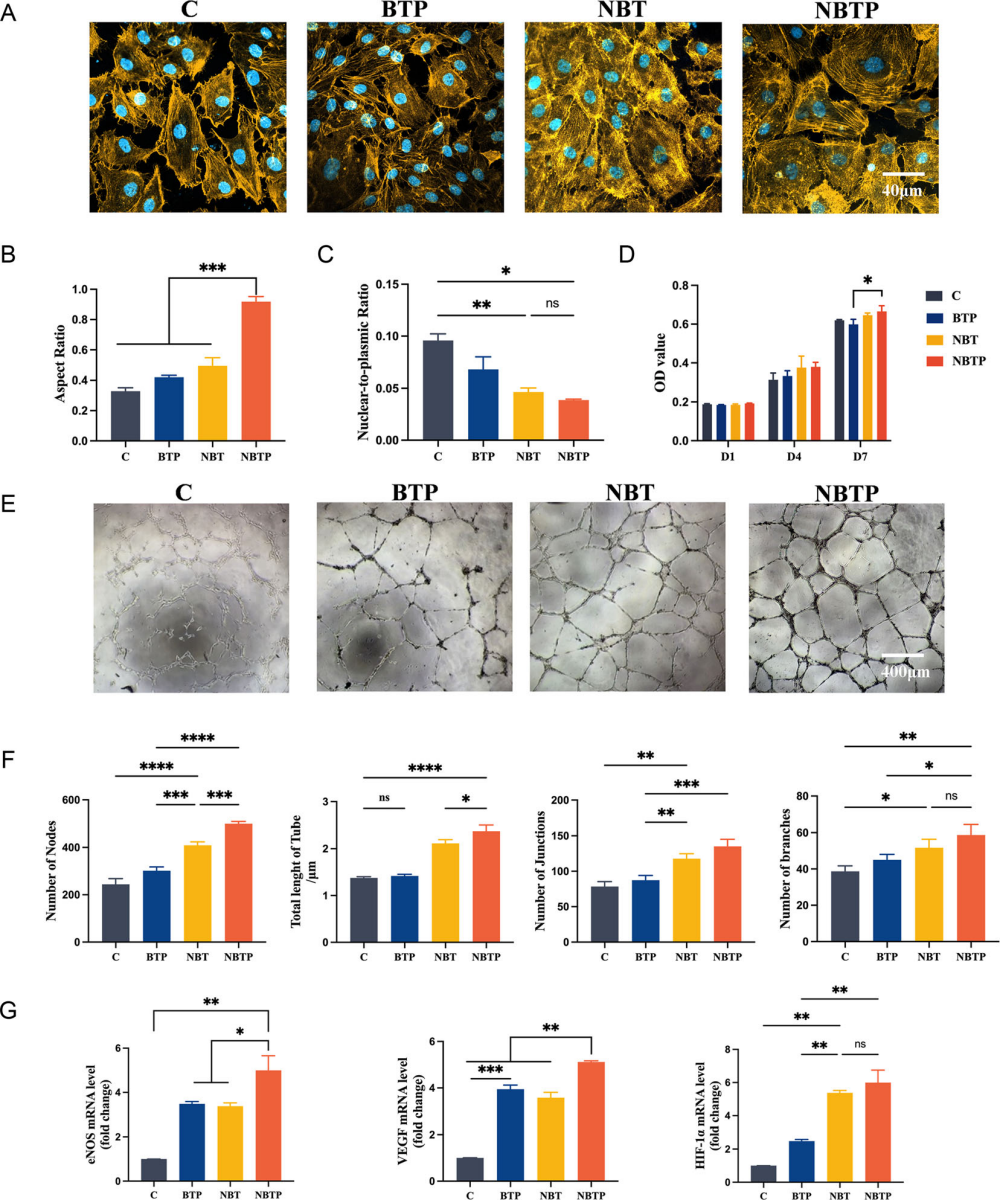

**Figure:**
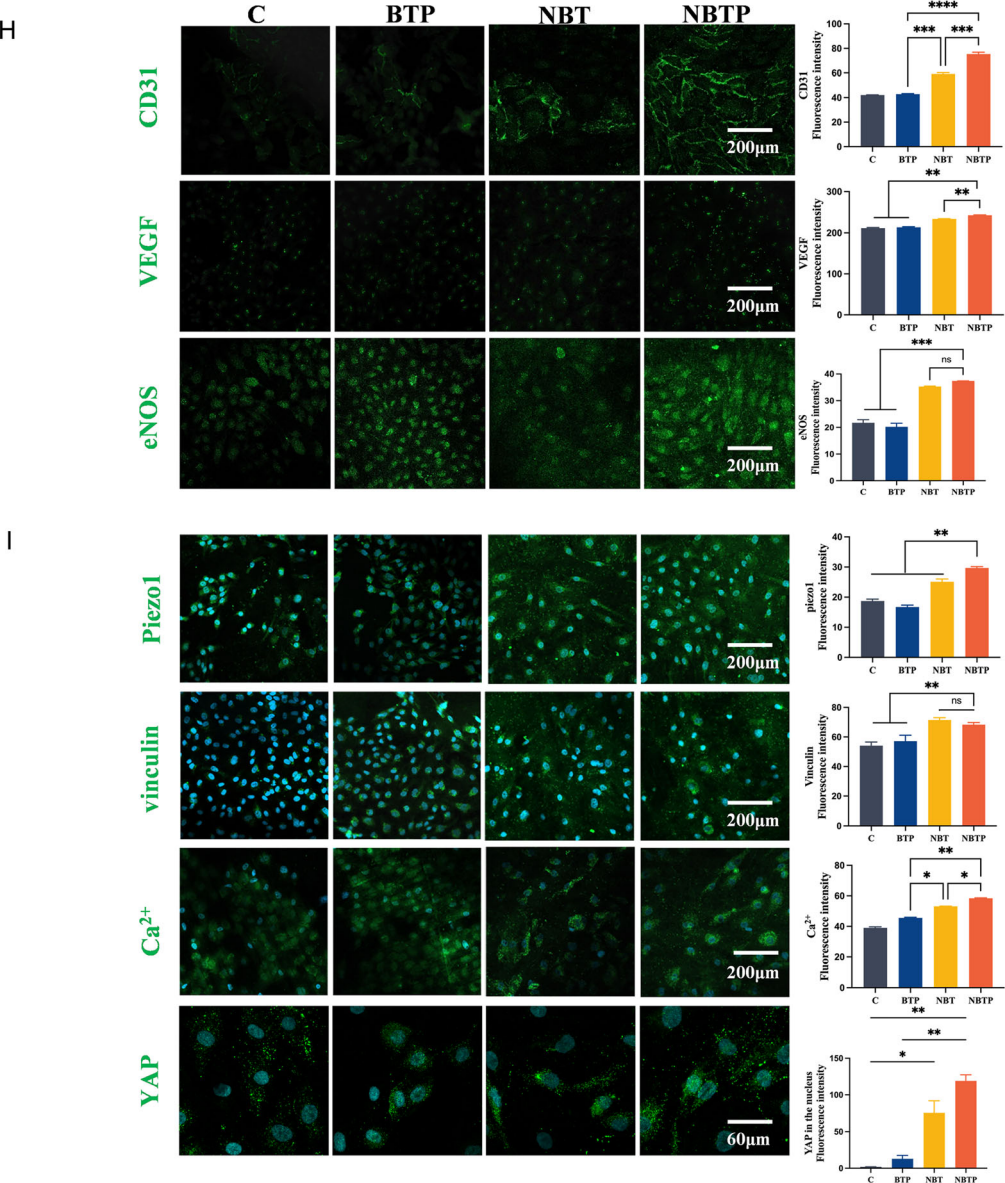

To investigate how mechanobiological–piezoelectric microenvironment enhances angiogenesis in HUVECs, we examined key molecular markers. Immunofluorescence staining revealed significantly higher Piezo1 and vinculin expression, along with elevated intracellular Ca^2+^ levels in the NBTP group compared to others (Figure 7I). YAP showed strong nuclear accumulation in NBTP and NBT groups, particularly in the NBTP, while remaining predominantly cytoplasmic in Ti and BTP groups (Figure 7I). Piezo1, a mechanosensitive ion channel, mediates endothelial responses to mechanical stimuli.^[^ ^58^ ^]^ In vascular ECs, it detects shear stress—critical for blood flow regulation and vessel formation.^[^ ^59^ ^]^ Mechanical activation of Piezo1 triggers calcium influx, initiating signaling pathways that regulate migration, proliferation, and angiogenesis. Thus, Piezo1 maintains vascular homeostasis and supports vessel development and remodeling.^[^ ^60^, ^61^ ^]^ Vinculin, a load‐bearing linker protein, facilitates mechanotransduction through tail domain interactions with filamentous actin.^[^ ^62^ ^]^ YAP, a mechanosensitive transcriptional factor, regulates growth and survival genes upon nuclear translocation.^[^ ^63^, ^64^ ^]^ In ECs, nuclear YAP accumulation promotes angiogenesis by stimulating endothelial sprouting and vascular regeneration.^[^ ^65^, ^66^ ^]^ Our findings demonstrate that increased vinculin expression and YAP nuclear localization in the NBTP and NBT groups indicated cytoskeleton reorganization and biomechanical force induction by the nanotopography. Together, these results indicate that the built‐in electric field improves mechanical stress‐mediated angiogenesis in HUVECs through Piezo1/Ca^2+^ signaling.

### 2.7 In Vitro Angiogenic Regulation of BMSCs

During bone‐defect regeneration, MSCs–HUVEC crosstalk is crucial for vascularized osteogenesis.^[^ ^67^ ^]^ To identify MSC‐derived angiogenic signals, we analyzed angiogenesis‐associated gene expression profiles using RT‐qPCR and measured paracrine protein factors by Enzyme‐Linked Immunosorbent Assay (ELISA).

**Figure 8:**
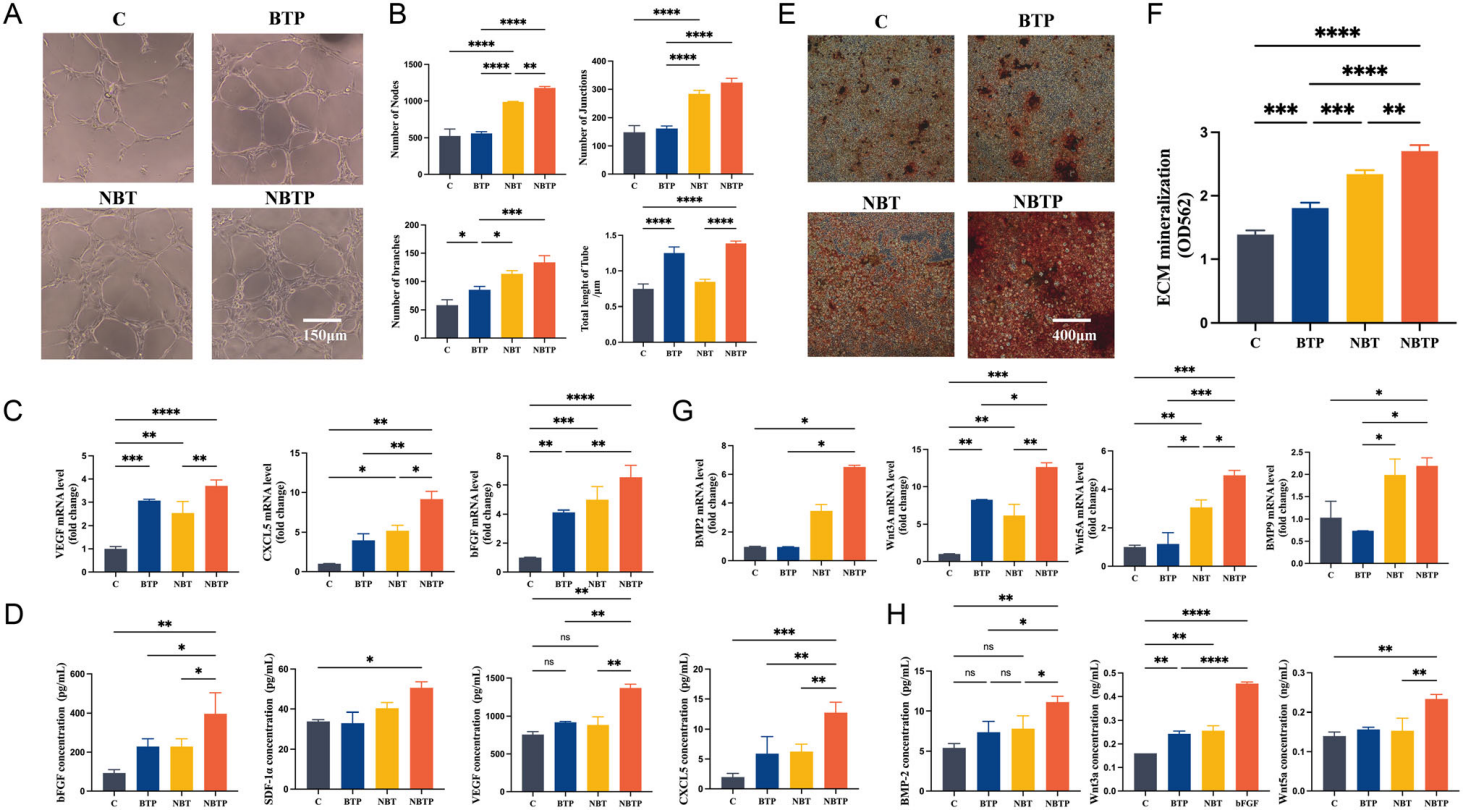
Crosstalk between BMSCs and HUVECs mediated by orchestrated mechanobiological–piezoelectric microenvironment. A) Tube formation of HUVECs cultured in BMSC‐conditioned media on different surfaces for 7 days. B) Semiquantitative analysis of tube formation parameters. C) mRNA expression of angiogenesis‐related genes (eNOS, VEGF, HIF‐1α) in BMSCs. D) Secretion levels of angiogenic factors (bFGF, DS‐1α, VEGF, CXCL5) from BMSCs by ELISA. E) ARS staining of BMSCs cocultured with HUVECs for 7 days. F) Semiquantitative analysis of calcium deposition. G) mRNA expression of osteogenic genes (BMP2, BMP4, Wnt3a, Wnt5a, BMP9) in HUVECs. H) Secretion levels of osteogenic factors (Wnt3a, Wnt5a, BMP2) from HUVECs by ELISA. ANOVA followed by Tukey's post hoc test was performed for statistical analysis (error bar: ±SD; *n* = 3; *p < 0.05, **p < 0.01, ***p < 0.001, ****p < 0.0001).

RT‐qPCR analysis indicated significant higher mRNA expression of angiogenesis‐associated factors (VEGF, bFGF, CXCL5) in the NBTP group compared to that in NBT, BTP, and C groups (*p *< 0.05; **Figure** **8**C). While NBT and BTP groups exhibited comparable expression levels (*p* > 0.05), both exceeded C levels (*p *< 0.05; Figure 8A). ELISA showed that BMSCs on NBTP secreted approximately twice as much bFGF, VEGF, and CXCL5 versus other surfaces, with BTP and NBT exhibiting intermediate levels above pure Ti (Figure 8D). In tube formation assays using BMSC‐conditioned media, HUVECs treated with NBTP supernatants exhibited the most robust angiogenesis, with significantly increased nodes, branches, junctions, and total tube length compared to other groups (*p *< 0.05; Figure 8B). NBT‐conditioned media showed generally stronger proangiogenic effects than BTP. These results indicate that the mechanobiological–piezoelectric microenvironment enhances BMSC‐mediated angiogenesis, particularly through doubled secretion of VEGF, bFGF, and CXCL5.

The transcriptome sequencing results identified CXCL5 as a key signaling factor in MSC–HUVEC communication (Figure 4A). Beyond its established role in bone regeneration, CXCL5 binds to the C–X–C motif chemokine receptor 2 (CXCR2), promoting neutrophil recruitment/activation and enhancing angiogenic processes—critical for both wound healing and tumor progression.^[^ ^41^, ^68^, ^69^ ^]^ Xu et al. demonstrated that in MSCs, PI3K/AKT‐mediated AKT phosphorylation upregulated CXCL5 expression by promoting CCL7‐dependent formation of KLF5–P300 complex on the CXCL5 promoter,^[^ ^41^ ^]^ consistent with our findings. Additionally, MSCs stimulate angiogenesis through the paracrine secretion of various factors including VEGF, bFGF, and SDF1.^[^ ^70^, ^71^, ^72^ ^]^

### 2.8 In Vitro Osteogenic Regulation of HUVECs

Beyond providing essential blood flow supply and metabolic transport for bone regeneration, HUVECs also promote osteogenesis through paracrine secretion of osteogenic factors.^[^ ^1^ ^]^ To identify HUVEC‐derived signals influencing BMSC osteogenic differentiation and bone regeneration, we analyzed osteogenesis‐related gene expression (RT‐qPCR) and paracrine protein secretion (ELISA).

RT‐qPCR analysis revealed a significantly higher mRNA expression of osteogenic factors (BMP2, WNT3a, BMP4, BMP9) in the NBTP group versus other groups (*p *< 0.05; Figure 8G). ELISA confirmed corresponding increases in BMP2, Wnt3a, and Wnt5a protein secretion, with Wnt3a levels in NBTP being approximately double those of other groups (Figure 8H). ECM mineralization assays revealed that BMSCs cultured with NBTP HUVEC‐conditioned media exhibited robust calcium accumulation, while C group media produced minimal deposition (Figure 8E,F). These findings demonstrate that the mechanobiological–piezoelectric microenvironment enhances BMSC osteogenic differentiation through HUVEC‐mediated paracrine secretion of Wnt3a, BMP2, and Wnt5a.

BMPs play a critical role in bone formation and regeneration.^[^ ^73^ ^]^ BMP2 and BMP7 are crucial for the initiation of osteogenic differentiation, promotion of BMSC commitment to the osteoblast lineage, and chondrogenesis. These factors have become standard therapeutic agents in orthopedic applications, including the treatment of nonunion fractures, spinal surgeries, and oral maxillofacial procedures.^[^ ^74^ ^]^ BMP9 represents one of the most potent osteogenic factors. The work by Muhammad Subhan Amir and colleagues highlighted its significant role in angiogenic ossification. In addition to its robust osteogenic capabilities, BMP9 enhances HIF‐1α regulation in osteoblasts through the PI3K–AKT pathway, subsequently promoting angiogenesis and augmenting angiogenic ossification.^[^ ^75^ ^]^ Wnt3a, a key component of the Wnt/β‐catenin signaling pathway, is secreted by ECs and plays an instrumental role in coordinating angiogenesis and osteogenesis.^[^ ^76^ ^]^ These findings suggest that the mechanobiological–piezoelectric microenvironment provided by the nanostructure and electrical signals of NBTP directly regulates intrinsic BMSC and EC functions and also activates their crosstalk through paracrine signaling. Together, these effects promote bone formation and enhance angiogenic ossification.

### 2.9 Restored Mechanobiological and Piezoelectric Microenvironment Enhances In Vivo Angiogenesis

**Figure 9:**
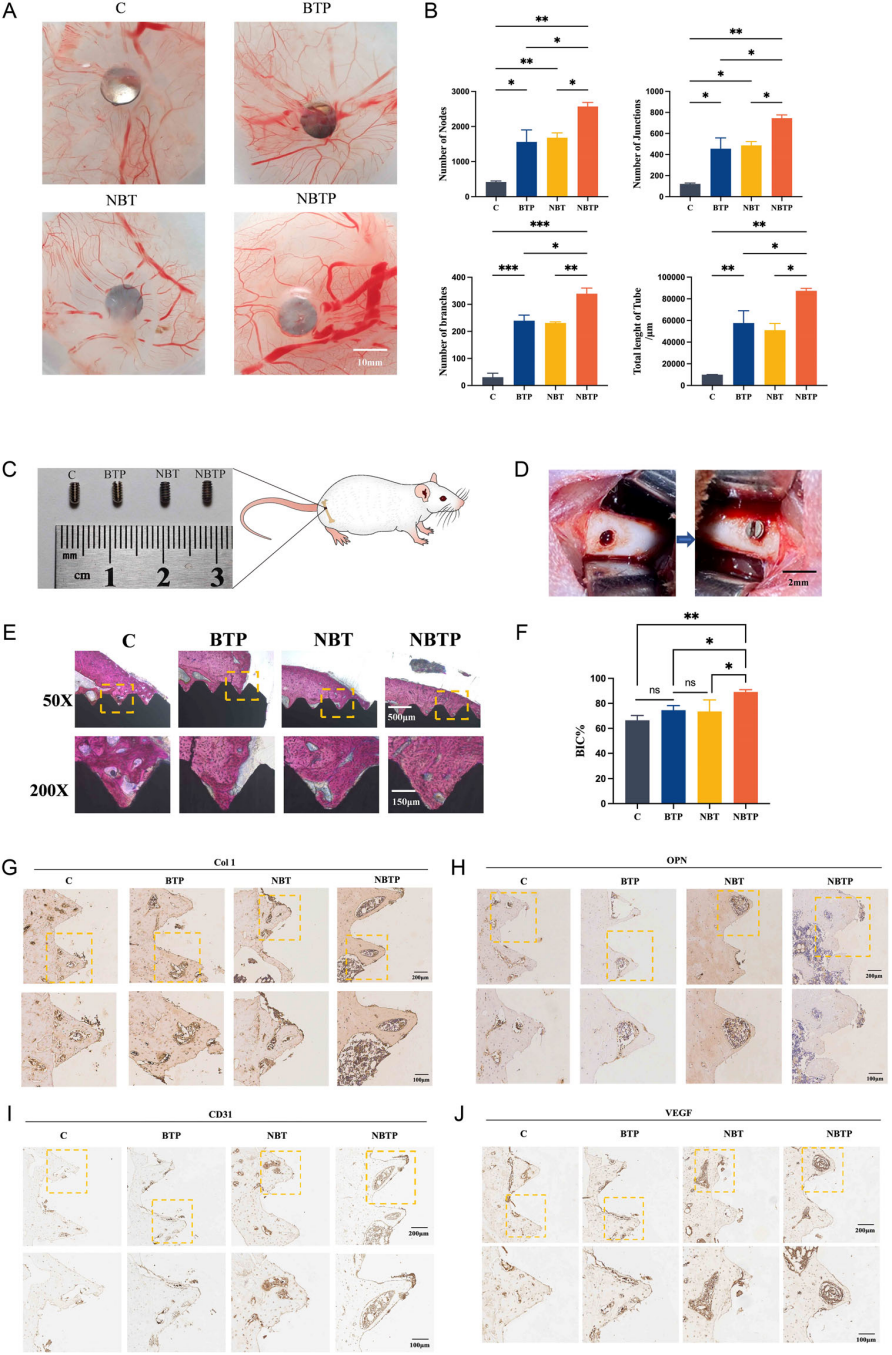
Enhanced angiogenesis through restored mechanobiological and piezoelectric microenvironment in vivo. A) CAM assay showing vascular networks formed around different implants. B) Semiquantitative analysis of vascular parameters from the CAM assay. C) Macroscopic analysis of implant size, shape, and morphology. D) Surgical site showing implant placement in rat femur. E) Methylene blue/basic fuchsin‐stained sections at 4 weeks postimplantation (new bone=red, fibrous tissue=blue, implants=black). F) Quantitative analysis of BIC% at 4 weeks. G) Immunohistochemical staining of Col1 in peri‐implant tissue at 4 weeks. H) Immunohistochemical staining of OPN in peri‐implant tissue at 4 weeks. I) Immunohistochemical staining of CD31 in peri‐implant tissue at 4 weeks. J) Immunohistochemical staining of VEGF in peri‐implant tissue at 4 weeks. Target proteins appear deep brown. ANOVA followed by Tukey's post hoc test was performed for statistical analysis (error bar: ±SD; *n *= 3; *p < 0.05, **p < 0.01, ***p < 0.001).

To evaluate the angiogenic potential of the materials in vivo, we used the chick CAM model. This system features rapidly expanding CAM structures that form dense vascular networks for efficient gas and waste exchange. The CAM model offers several advantages: simplicity, rapid results, low cost, short experimental duration, and exemption from ethics committee approval requirements for animal experimentation.^[^ ^77^, ^78^ ^]^ In our study, pathogen‐free fertilized chicken embryos were incubated for 5 days with Ti, BTP, NBT, or NBTP. CAM imaging showed that NBTP induced significantly more vascular branches and greater vascular density than other groups, forming an extensive, densely packed network. Both NBT and BTP groups showed similar proangiogenic effects, outperforming the Ti group (**Figure** **9**A). Semiquantitative analysis demonstrated that NBTP significantly increased in vessel length, junctions, and nodes compared to Ti (Figure 9B). These results confirm that the mechanobiological–piezoelectric microenvironment significantly enhances in vivo angiogenesis.

### 2.10 Restored Mechanobiological and Piezoelectric Microenvironment Enhances Vascularized Osteogenesis in Rats

Having successfully validated the excellent osteogenic differentiation‐promoting performance of NBTP in vitro, we further examined its bone integration capacity using a rat femoral defect model. A circular defect (2.0 mm diameter) was created in the rat femur. Then, Ti implants coated with NBTP, NBT, and BTP were inserted into the drilled hole (Figure 9C,D). Micro‐CT three‐dimensional reconstruction revealed generally greater bone formation around NBTP implants compared to other groups (Figure S2, Supporting Information). At 4 weeks postimplantation, VG staining demonstrated that NBTP‐coated implants showed increased new bone contact with the implant surface compared with other groups, promoting both osteoinduction and osteointegration (Figure 9E). Specifically, Ti implants were surrounded by more fibrous tissues (blue‐stained areas) with minimal new bone formation at the bone–implant interface, while NBTP implants showed continuous bone attachment that significantly increased the bone–implant contact (BIC) percentage. These results demonstrate that NBTP implants restoring the mechanobiological and piezoelectric microenvironment can substantially enhance both the speed and quality of bone integration in vivo (Figure 9F).

Immunohistochemical staining revealed significant modulation in the expression levels of bone‐related factors OPN and Col1. OPN plays a pivotal role in bone metabolism and remodeling. This protein is ubiquitously expressed across diverse cell types and tissues, with multiple regulatory roles in bone‐related pathophysiology. It critically modulates bone turnover and mineral density, significantly influencing bone formation and reconstruction.^[^ ^79^, ^80^ ^]^ Collagen type 1 (Col1) is essential for osteogenic differentiation and bone remodeling. As the most abundant bone matrix protein, Col1 maintains skeletal structural integrity. Its expression increases during early osteogenic differentiation, facilitating osteoblast maturation and bone matrix formation.^[^ ^81^, ^82^ ^]^ Notably, an elevated number of cells expressing OPN and Col1 was detected near the wedge‐shaped interface between the NBTP implant and bone (Figure 9G,H; S3 A,B, Supporting Information). In contrast, CD31 and VEGF expression near NBTP and NBT implants was significantly higher than in other groups (Figure 9I,J; S3C,D, Supporting Information). This localized increase in OPN, Col1, CD31, and VEGF expression suggests that the NBTP material primarily influenced the implant–bone interface, demonstrating the combined effects of electrophysiological and surface morphological microenvironments. Briefly, in vivo experiments verified that nanotopography played a dominant role in enhancing vascularized osseointegration. The inherent electrical activity further accelerates the topography‐mediated osseointegration by remodeling a mechanobiological–piezoelectric‐coupled microenvironment favorable for coupled angiogenesis and osteogenesis.

## 3 Conclusion

In this study, polarized BaTiO_3_ nanorod arrays (NBTP) were constructed on pure titanium substrates using alkali heat treatment, ion replacement reactions, and polarization. The nanotopography not only increased the hydrophilicity but also enhanced piezoelectric property surface potential, which was attributed to the significantly decreased Young's modulus. In vitro experiments showed that topography‐mediated mechanobiological remodeling enhanced the osteogenic potential of MSCs and angiogenic potential of ECs by activating Piezo2/Piezo1/Ca^2+^ signaling. The augmented electrical activity further improved mechanical‐stress‐mediated enhancement of MSC osteogenic potential by additionally activating Ca_v1.2_ in MSCs and further stimulating Piezo2/Piezo1 in MSCs/ECs, synergistically inducing Ca^2+^ influx. Microarray analysis of MSCs revealed the activation of the PI3K/AKT/mTOR/GSK3β signaling by mechanobiological–piezoelectric coupling. Both Ca^2+^ influx and PI3K/AKT/mTOR/GSK3β signaling activation, as well as the osteogenic superiority of NBTP, were attenuated when using Cav_1.2_ and Piezo2 blockers. Moreover, the coordination of these biophysical characteristics activated paracrine signaling‐mediated crosstalk between MSCs and ECs, indirectly increasing MSC angiogenic potential and EC osteogenic potential. Results from CAM and femur implantation studies confirmed that nanorod topography plays a dominant role in enhancing vascularized osseointegration. Inherent electrical activity further accelerates topography‐mediated osseointegration by remodeling a mechanobiological–piezoelectric‐coupled microenvironment favorable for coupled angiogenesis and osteogenesis. This study provides an approach for developing multifunctional bioactive coatings on metal implant surfaces and deepens the understanding of mechanobiological–piezoelectric regulation of vascularized osteogenesis. Our findings demonstrate that the precise engineering of nanoscale bioelectrical microenvironments could serve as a novel and critical strategy to enhance vascularized osteogenesis, with potential applications in vascularization‐impaired conditions such as diabetes mellitus and osteoradionecrosis.

## 4 Experimental Section

4.1

4.1.1

##### Fabrication and Characterization of BaTiO_*3*_ *Nanorod Arrays*: *Material preparation*

Potassium hydroxide (KOH, ACS, 98%), concentrated nitric acid, sodium hydroxide (NaOH, ACS, 98%), barium hydroxide octahydrate (Ba(OH)_2_·8H_2_O, AR, 99%), and hydrofluoric acid were obtained from Aladdin Co., Ltd. (Shanghai, China). Titanium sheets were utilized as the substrate material.

##### Fabrication and Characterization of BaTiO_*3*_ *Nanorod Arrays: Barium Titanate (BT) Coating synthesis*

BT was synthesized via hydrothermal treatment. Titanium plates were immersed in an aqueous solution containing 0.05 M Ba(OH)_2_·8H_2_O and 2 M KOH and then hydrothermally treated at 180 °C and for 8 h. Polarized BT (BTP) was subsequently obtained by applying 0.4 kV voltages during polarization.

##### Fabrication and Characterization of BaTiO_*3*_ *Nanorod Arrays: Sodium Titanate Nanorod Arrays Synthesis*

Titanium plates were first cleaned by sequential ultrasonic treatment with acetone, ethanol, and ultrapure water to remove surface impurities. Sodium titanate nanorod arrays were then grown on the titanium surfaces via alkali heat treatment with 2 M NaOH solution at 100 °C for 24 h. Finally, the samples were rinsed with deionized water to remove unreacted reagents.

##### Fabrication and Characterization of BaTiO_*3*_ *Nanorod Arrays: Barium Titanate Nanorod Array Coating Synthesis (NBT)*

The ion exchange reaction served as the primary method for NBT preparation. Sodium titanate nanorod arrays were hydrothermally treated in 0.05 M aqueous Ba(OH)_2_·8H_2_O solution at 210 °C for 8 h. After reaction, the unreacted Ba(OH)_2_·8H_2_O was removed by rinsing repeatedly with deionized water. Polarization was then performed by applying 0.4 kV for 5 min to obtain polarized NBTP bearing surface charge.

##### Fabrication and Characterization of BaTiO_*3*_ *Nanorod Arrays: Characterization of Material Properties*

The surface morphologies of all samples were characterized using field‐emission SEM (Carl Zeiss, Germany). Crystal composition and phase identification were analyzed by XRD (Empyrean, Panalytical, Netherlands). For NBT nanorods specifically, morphology, crystal structure, and elemental distribution were examined using TEM (JEM‐2100 F, JEOL, Japan). Titanium surface hydrophilicity was evaluated by water contact angle measurements (DSA‐XROLL; Beituo, China). Mechanical properties were assessed by measuring Young's modulus using atomic force microscopy (AFM; Dimension Edge, Bruker, USA). Piezoelectric coefficients (d_33_) and surface potentials of samples were measured using PFM and Kelvin probe force microscopy modes, respectively, of an AFM (Multimode 8, Bruker, USA).

##### Cell Culture and Seeding

BMSCs isolated from C57BL/6 mice were purchased from Cyagen Co. (USA). BMSCs were cultured in high‐glucose Dulbecco's modified Eagle's medium (DMEM) supplemented with 10% fetal bovine serum (FBS) and 1% penicillin–streptomycin. HUVECs were purchased from BIOSPECIES (China) and maintained in endothelial cell medium (ScienCell, USA) containing 5% FBS, 1% Endothelial Cell Growth Factor (ECGF), 50 U mL^−1^ of heparin sulfate, 1 μg mL^−1^ of hydrocortisone hemisuccinate, 50 μg mL^−1^ of ascorbic acid, and 1% penicillin–streptomycin. For experiments, BMSCs or HUVECs were cocultured onto Ti discs (pure Ti, BT, BTP, NBT, or NBTP) at 37 °C in a humidified 5% CO_2_ atmosphere. For differentiation studies, MSCs were cultured in osteogenic differentiation medium (Cyagen Co.).

##### Cell Proliferation, Morphology, and Cytoskeleton Assays of MSCs

BMSCs were seeded onto material surfaces at 5 × 10^4^ cells/well in complete medium to assess proliferative and cytotoxicity activities. Cell viability was quantified using CCK‐8 (Dojindo, Japan) after 1, 4, and 7 days of culture. Medium was changed every 2 days. At each time point, samples were rinsed with PBS and incubated with 10% CCK‐8 solution in fresh medium for 2 h (protected from light). The reaction solution (100 μL) was then transferred to a 96‐well plate, and absorbance was measured at 450 nm using a microplate reader. For morphological analysis, BMSCs were cultured on materials for 1 day, washed with PBS, and fixed with 4% paraformaldehyde. Cytoskeletal organization was visualized using Alexa Fluor‐conjugated phalloidin (for F‐acting staining), with 4′,6‐Diamidino‐2‐Phenylindole (DAPI) counterstaining for nuclei. Fluorescence images were acquired using a confocal laser‐scanning microscope (LSM980; ZEISS, Germany).

##### In Vitro Osteogenic Differentiation Evaluation

BMSCs were cultured under osteogenic conditions for 14 and 21 days prior to Alizarin Red S (Cyagen Co.) staining. After PBS washing and 4% paraformaldehyde fixation, calcium nodules within the ECM were stained and imaged using a stereomicroscope (M205A; Leica, Germany). For semiquantitative analysis, stained discs were dissolved in 10% cetylpyridinium chloride (Sigma), and absorbance was measured at 562 nm.

##### Detection of Calcium Fluxes and Osteogenic Regulatory Pathways of MSCs: Intracellular Calcium Ion analysis

BMSCs were seeded on test materials in 48‐well plates at 5 × 10^4^ cells/well. After 1 and 7 days of culture, cells were incubated with 1 μM Fluo‐4 AM (dissolved in PBS) for 1 h at 37 °C protected from light. Samples were then transferred to the dishes for imaging using a laser scanning confocal microscope (LSM980, ZEISS, Germany). Intracellular calcium localization and concentration were analyzed by quantifying Fluo‐4 AM fluorescence intensity using ImageJ software (v1.53a; National Institutes of Health, USA).

##### Detection of Calcium Fluxes and Osteogenic Regulatory Pathways of MSCs: Microarray Analysis

Total RNA was extracted from cells cultured on control (C), BT, BTP, NBT, and NBTP surfaces using TRIzol reagent (Gibco‐BRL, Gaithersburg, MD, USA) following the manufacturer's instructions. RNA purification was performed using magnetic bead‐based methods. Sequencing was conducted on an Illumina platform (Illumina, USA). Differential gene expression analysis was performed using edgeR, pathway enrichment analysis conducted through the KEGG database. PCA was implemented using the factoextra R package.

##### Detection of Calcium Fluxes and Osteogenic Regulatory Pathways of MSCs: Quantitative Real‐Time Polymerase Chain Reaction Analysis

The expression levels of Ca_v1.2_, Piezo2, PI3KCA, AKT1, and AKT3 and the osteogenic genes Runx2, OCN, and Col1 were quantified by RT‐qPCR. Total RNA was extracted using TRIzol reagent followed by cDNA synthesis with a reverse transcription kit (Takara Bio Inc., Japan). RNA concentration was normalized using a Nanodrop 2000 spectrophotometer (Thermo Scientific, USA). RT‐qPCR was performed using SYBR Green master mix (Invitrogen) under the following conditions: 95 °C for 30 s, then 38 cycles of 95 °C for 5 s and 60 °C for 30 s. Primer sequences for Ca_v1.2_ and Piezo 2 are listed in Table S1, Supporting Information. Glyceraldehyde 3‐Phosphate Dehydrogenase (GAPDH) served as the housekeeping gene for normalization, and relative gene expression was calculated using the 2^−ΔΔCt^ method.

##### Detection of Calcium Fluxes and Osteogenic Regulatory Pathways of MSCs: Western Blot Assay

Total cellular proteins were extracted from BMSCs after 48 h of culture using ice‐cold lysis buffer for 30 min. Protein concentration of proteins was determined using a Bicinchoninic Acid Assay (BCA) protein detection kit (KWBIO, China). Equal protein amounts (40 μg) were separated by Sodium Dodecyl Sulfate‐Polyacrylamide Gel Electrophoresis (SDS‐PAGE) gels and transferred to nitrocellulose membranes. Membranes were blocked in 5% nonfat milk in Tris‐buffered saline‐Tween buffer (TBST) and then incubated overnight at 4 °C with primary antibodies from Affinity Biosciences (China): GAPDH (T0004), OPN (AF0227), OCN (DF12303), PI3K (AF6241), phospho‐PI3K (P‐PI3K; AF3242), phospho‐AKT (P‐AKT; AF0016), and AKT (AF6261). After TBST washes three times, membranes were incubated with Horseradish Peroxidase (HRP)‐conjugated secondary antibodies (goat antirabbit, EM35111‐01; goat antimouse EM35110‐02; Beijing Emarbio Science & Technology Company, China) for 1 h. Signals were detected using a ChemiDoc Imaging System (Bio‐Rad, USA), and the density of each band was quantified with ImageJ software.

##### Effect of Calcium Channel Blocking on Intracellular Calcium Dynamics, PI3K/AKT Pathway Expression, and Osteogenic Potential of MSCs on NBTP Surface

To investigate the roles of the L‐type calcium channels (blocked by nifedipine) and Piezo2 (inhibited by GsMTx4) in calcium signaling, PI3K/AKT pathway expression, and the enhanced osteogenic potential of MSCs on NBTP surfaces, we employed these specific pharmacological inhibitors in our experimental design.

The NBTP samples were randomly divided into four groups: NBTP‐G, NBTP‐N, NBTP‐D, and NBTP. Polished pure Ti samples served as the control (C) group. The NBTP‐G group was cultured in complete medium containing 2.5 μM GsMTx4 (MedChemExpress, USA), while the NBTP‐N group was cultured received with 10 μM nifedipine (MedChemExpress, USA). The NBTP‐D group was cultured in medium containing both 2.5 μM GsMTx4 and 10 μM nifedipine. For comparison, the NBTP and C groups were maintained in complete medium alone. After 24 h of culture, calcium ion dynamics were analyzed by using a fluorescent probe. Expression levels of Ca_v1.2_, Piezo2, PI3KCA, AKT1, AKT3, and osteogenic marker genes were quantified by RT‐qPCR.

##### In Vitro Angiogenic Behaviors of HUVECs: HUVEC Morphology

To observe HUVEC morphology, cells (4 × 10^4^ cells cm^−2^) were seeded onto substrates in 24‐well plates and cultured for 24 h. Cells were then fixed with 4% paraformaldehyde (30 min, room temperature) and permeabilized with 0.2% Triton X‐100 (20 min, room temperature). For cytoskeletal (F‐actin) visualization, samples were stained with rhodamine‐phalloidin (5 U mL^−1^; Beyotime, China) overnight at 4 °C for F‐actin staining followed by nuclear counterstaining with 2‐(4‐amidinophenyl)‐1H‐indole‐6‐carboxamidine (DAPI; Beyotime, China) for 5 min at room temperature. Imaging was performed using an inverted fluorescent microscope.

##### In Vitro Angiogenic Behaviors of HUVECs: Cell Viability of HUVECs

HUVECs were seeded onto substrates at a density of 4 × 10^4^ cells cm^−2^. Cell proliferation was assessed using the CCK‐8 assay after 1, 4 and 7 days of culture.

##### In Vitro Angiogenic Behaviors of HUVECs: In Vitro Angiogenesis

The formation of vascular‐like structures by HUVECs on different substrates was evaluated using growth factor‐reduced Matrigel Matrix (Corning, USA). Briefly, 200 μL of Matrigel was polymerized in each well and incubated for 30 min at 37 °C, 5% CO_2_. HUVECs were cultured on Ti, BTP, NBT, and NBTP surfaces for 7 h; then, cells were trypsinized, resuspended in DMEM with 1% FBS, and seeded at 5 × 10^4^ cells/well. After 12 h of incubation, tube formation was observed using an inverted microscope. Three to five random fields per sample were analyzed for tube counting.

##### In Vitro Angiogenic Behaviors of HUVECs: mRNA and Protein Expression of Angiogenic Factors

HUVECs (2 × 104 cells cm^−2^) were seeded onto substrates in 24‐well plates. After 3 days of incubation, RT‐qPCR was performed to detect the mRNA expression of angiogenic factors including eNOS, VEGF, and HIF‐1α according to the method described above. Gene expression levels were normalized to that of the endogenous housekeeping gene, GADPH. Immunocytochemical staining was used to detect the expression of angiogenic factors, including CD31, VEGF, eNOS, vinculin, YAP, and Piezo1. Briefly, adherent HUVECs were fixed in 4% w/v paraformaldehyde, incubated with primary antibodies, and stained with Fluorescein Isothiocyanate (FITC)‐conjugated goat antirabbit secondary antibody. Finally, the cell nuclei were labeled with DAPI (Beyotime, China). Images were captured using a confocal laser scanning microscope. Primary antibodies utilized included rabbit polyclonal antibody to CD31 (#AF6191, Affinity, China), anti‐VEGFA (#AF5131, Affinity, China), anti‐eNOS (#AF0096, Affinity, China), anti‐Piezo1 (#DF12083, Affinity, China), anti‐Vinculin (#AF5122, Affinity, China), and anti‐YAP (#AF6328, Affinity, China). Goat antirabbit (ab150081, Affinity, China) and goat antimouse (ab150115, Abcam, Cambridgeshire, UK) were utilized as secondary antibodies. Intracellular calcium localization and concentration in HUVECs were analyzed using the fluorescent probe Fluo‐4 AM according to the method described above.

##### Osteogenic Regulation of HUVECs: mRNA and Protein Expression of Osteogenic Factors

HUVECs (4 × 10^4^ cells cm^−2^) were seeded onto substrates in 24‐well plates and cultured for 3 days. Total RNA was extracted for RT‐qPCR analysis of osteogenic gene expression as previously described. Protein expression of osteogenic factors was quantified by ELISA according to the manufacturer's protocols.

##### Osteogenic Regulation of HUVECs

An indirect coculture system was established to investigate HUVEC‐mediated regulation of MSC osteogenic differentiation. HUVECs (4 × 10^4^ cells cm^−2^) were seeded onto substrates in 24‐well plates. After 48 h of incubation, the conditioned medium was collected by harvesting the HUVEC supernatant and mixing 1:1 with osteogenic differentiation medium. MSCs were then cultured in this conditioned medium for 21 days, and ECM mineralization was assessed as previously described.

##### Angiogenic Regulation of MSCs: mRNA and Protein Expression of Angiogenic Factors

BMSCs (4 × 10^4^ cells cm^−2^) were seeded onto substrates in 24‐well plates and cultured for 3 days. Total RNA was extracted for RT‐qPCR analysis of angiogenic gene expression as previously described. Protein levels of angiogenic factors were quantified using ELISA according to the manufacturer's protocols.

##### Angiogenic Regulation of MSCs

An indirect coculture system was established to investigate MSC‐mediated regulation of HUVEC angiogenic activity. MSCs (4 × 10^4^ cells cm^−2^) were seeded onto substrates in 24‐well plates. After 48 h of incubation, the conditioned medium was prepared by harvesting MSC supernatants and mixing 1:1 with complete MSC medium. HUVECs were then cultured in this conditioned medium on growth factor‐reduced Matrigel matrix for 24 h, and tube formation was assessed as previously described.

##### In Vivo *Evaluation of Vascularized Osteogenesis: CAM Assays*

Pathogen‐free fertilized chicken eggs (E5 stage) were obtained from the Poultry Center of South China Agricultural University and preincubated for 1 d at 37 °C with a 60% relative humidity. The CAM was exposed by creating a window at the pointed end of each egg. Material discs (4.2 mm diameter) were fabricated using a sterile biopsy punch and placed inverted onto vascularized CAM regions. The CAM surface was moistened with a sterile saline solution to maintain hydration. After 5 days of incubation, bright‐field images of the material and surrounding vasculature were captured using a Canon EOS 6D DSLR camera (6D, Canon, Japan) in macro mode. ImageJ software was used to quantify angiogenesis.

##### In Vivo Evaluation of Vascularized Osteogenesis: Surgical Procedures

Twenty‐four male Sprague‐Dawley (SD) rats (220–250 g) were purchased from the Animal Experiment Center, East Campus, Sun Yat‐sen University. All animal experiments were approved by the Institutional Animal Care and Use Committee of Sun Yat‐sen University (2 022 001 070) and conducted in accordance with the established ethical guidelines of the committee. A bilateral distal femur defect model was established to evaluate the osteogenic properties of Ti, BTP, NBT, and NBTP. Rats were anesthetized with intraperitoneal pentobarbital (30 mg kg^−1^) and received local lidocaine at the surgical site. Under aseptic conditions, distal femurs on both sides were exposed and four groups of titanium implants were randomly inserted into the prepared holes until the screw threads were fully seated in the bone cortex. After 4 weeks, implants and surrounding bone were harvested and fixed in 4% paraformaldehyde for subsequent experiments.

##### In Vivo Evaluation of Vascularized Osteogenesis: Micro‐CT

After 24 h of fixation in 4% paraformaldehyde, the newly formed bone in the femoral defect area was scanned using a micro‐CT imaging. 3D reconstructions were generated and analyzed using micro‐CT software.

##### In Vivo Evaluation of Vascularized Osteogenesis: Histological Evaluation

The newly formed bone was characterized using methylene blue/acid fuchsin and immunohistochemical staining. Tissue processing and sectioning were performed as described in our previous study.^[^ ^83^ ^]^ Briefly, methyl methacrylate‐embedded tissue samples were sectioned at 5–20 μm thickness after dehydration in a graded ethanol series (70%–100%). The sections were then stained with methylene blue/acid fuchsin (Merck) and observed under an inverted fluorescent microscope (Axio, ZEISS, Germany) to calculate the percentage of BIC% using ImageJ analysis software. For immunohistochemical staining, decalcified sections were incubated with rabbit anti‐OPN (0026 R, Bioss, China), rabbit anti‐Collagen I (0578 R, Bioss, China), and HRP‐conjugated goat antirabbit antibodies (GB23303, Servicebio, China) for immunohistochemical analysis. The sections were examined histomorphometrically using a ScanScope XT tissue slide scanner (Aperio, Leica Biosystems, Buffalo Grove, IL, USA). The mean optical density and cumulative optical density of the stained proteins were analyzed using ImagePro Plus software.

##### Statistical Analysis

All data were expressed as mean ± standard deviation (SD). Statistical analyses were performed using GraphPad Prism software (version 9.0) via a one‐way analysis of variance (ANOVA), followed by Tukey's post hoc test. Significance thresholds were set as *p  <  0.05, **p  <  0.01, or ***p  <  0.001.

## Conflict of Interest

The authors declare no conflict of interest.

## Supporting information

Supplementary Material

## Data Availability

The data that support the findings of this study are available from the corresponding author upon reasonable request.
