# Supplementary material for: Built‐In Electric Field Accelerates Nanotopography‐Mediated Enhancement of Vascularized Osseointegration via Cav1.2/Piezo/Ca2+/PI3K Signaling
Source: Small Sci. 2025 Jul 14;5(10):2500095. doi: 10.1002/smsc.202500095 (PMC12499458; doi:10.1002/smsc.202500095)
Supplement: Supplementary file 1 — Supplementary Material [file SMSC-5-2500095-s001.pdf]

Built-in Electric Field Accelerates Nanotopography-Mediated Enhancement of Vascularized Osseointegration via $Ca_{v1.2}$/Piezo/Ca^{2+}/PI3K Signaling

Jingyan Huang [a, 1], Dongheng Lu [a, 1], Cairong Xiao [b], Jiezhong Guan [a], Xiaoshuang Wang[a], Changhao Li[a], Peng Yu [b, *], Yan Wang [a, *]

[a] Hospital of Stomatology, Guanghua School of Stomatology, Sun Yat-sen University, Guangdong Provincial Key Laboratory of Stomatology, Guangzhou 510055, China

[b] School of Materials Science and Engineering, National Engineering Research Center for Tissue Restoration and Reconstruction, South China University of Technology, Guangzhou 510641, China

* Corresponding authors.

E-mail addresses: imyup@scut.edu.cn (P. Yu), wangyan9@mail.sysu.edu.cn (Y. Wang).

[1] These authors contributed equally.

Table S1. Primer sequences

Gene	Forward primer sequence （5’-3’）	Reverse primer sequence(5’-3’)
**Runx2**	GACTGTGGTTACCGTCATGGC	ACTTGGTTTTTCATAACAGCGGA
**COL-1**	GCTCCTCTTAGGGGCCACT	ATTGGGGACCCTTAGGCCAT
**OCN**	GGTAGTGAACAGACTCCGGC	GGCGGTCTTCAAGCCATACT
**Cav1.2**	TACCGTCAGTTCCACACAGC	CTTCAGAGTCAGGCAGAGCA
**Piezo2**	GTGGTATGCAACCCAGTACCC	GGCCATTCTCTATGGGCAGG
**GAPDH**	AGGTCGGTGTGAACGGATTTG	TGTAGACCATGTAGTTGAGGTCA
**mTOR**	CTGATCCCAACGAGCTAGTTC	GGTCTTTGCAGTACTTGTCATG
**GSK3β**	AAGAGTGCAGGTGTGTCTCG	GAAGTGCAAAGCAGCTGGTC
**FOXO1**	ATCACCAAGGCCATCGAGAGC	ACTGTTGTTGTCCATGGACGC
**AKT1**	CTGCCCTTCTACAACCAGGA	CATACACATCCTGCCACACG
**AKT2**	CTTCGGCAAGGTCATTCTGG	TTGAGGGCTGTAAGGAAGGG
**AKT3**	AGCCCAACCTCACAGATTGA	GTGCCACTTCATCCTTTGCA
**PI3KCA**	CTAGGATTCGTGGGGGCATC	TGCAGAGGGTCAGAGCAATG
**Human-HIF-1α**	TTTTCGTTGGGTGAGGGGAG	GCTTTAACTTTGCTGGCCCC
**Human-VEGF**	TACCTCCACCATGCCAAGTG	ATGATTCTGCCCTCCTCCTTC
**Human-eNOS**	CGGCATCACCAGGAAGAAGA	GCCATCACCGTGCCCAT
**Human-BMP2**	ACTCGAAATTCCCCGTGACC	CCACTTCCACCACGAATCCA
**Human-BMP9**	GCGGGTAAACTTCGAGGACA	CTGCACGATAGCGTGTTTCG
**Human-Wnt3a**	ACTTTTGTGAGCCCAACCCA	TTCTCCGTCCTCGTGTTGTG
**Human-Wnt5a**	CCGAGCGGAGAGGGTTATC	TGCACATGTAGCCATCAGAACA
**Human-GAPDH**	GAAGGTGAAGGTCGGAGT	GAAGATGGTGATGGGATTTC
**bFGF**	CTCGGCTTCAGGAAGAGTCC	GTCCCGTTTTGGATCCGAGT
**CXCL5**	TCCACACCTCCTCCAGCATA	CCGTGGGTGGAGAGAATCAG
**VEGF**	ACTTCTGAGGGGCCTAGGAG	GGCAGAGCTGAGTGTTAGCA

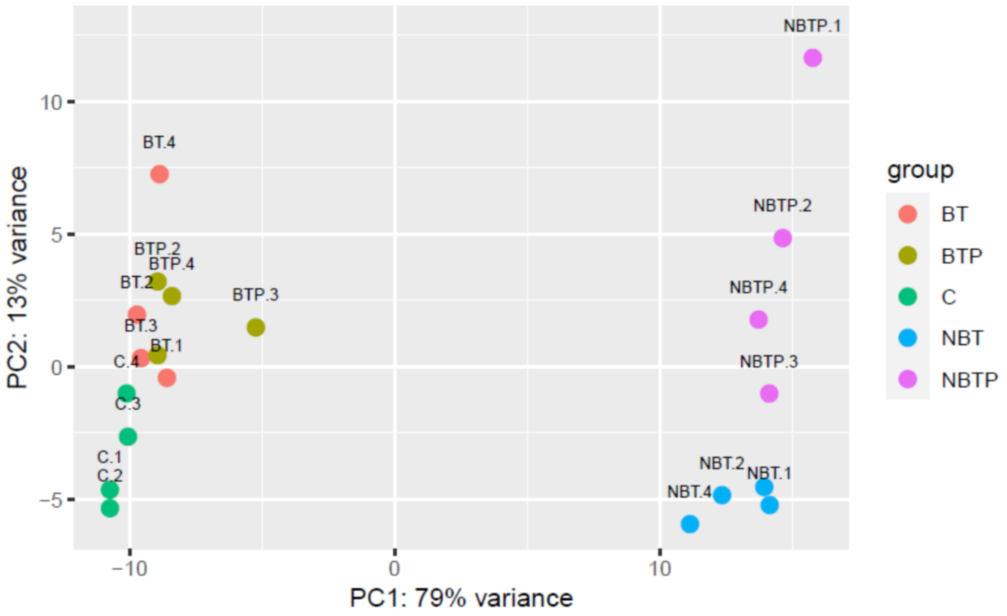

Figure S1. Principal component analysis of genes in MSCs from the C, BT, BTP, NBT and NBTP groups.

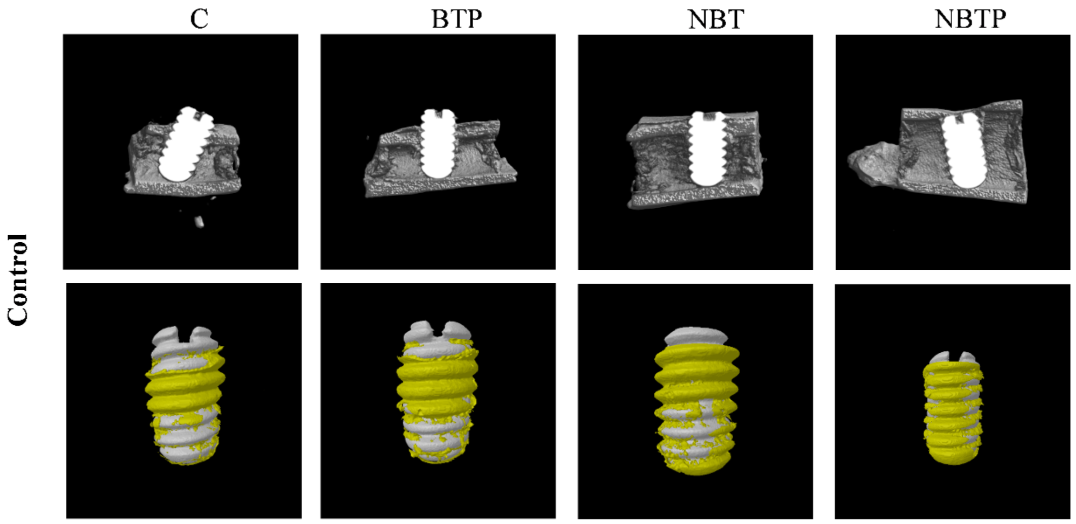

Figure S2. 2D and 3D reconstruction of the longitudinal profile of the new bone around the implants in rat femurs by MicroCT scanning.

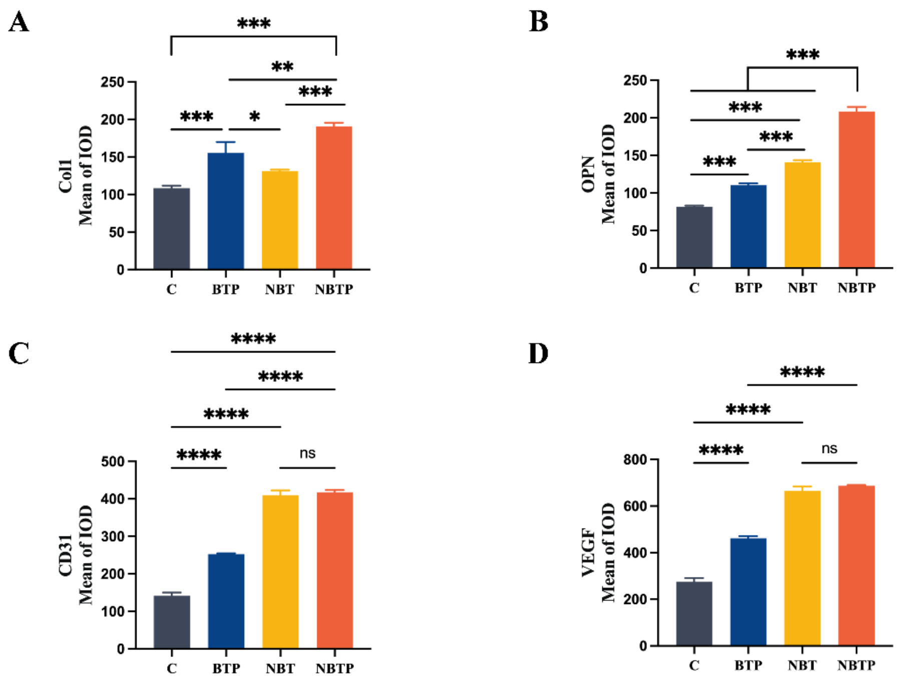

Figure S3. Quantitative analysis of osteogenic proteins Col1 and OPN (A&B) and angiogenic proteins CD31 and VEGF(C&D) in bone tissues around titanium implants by immunohistochemical staining. ANOVA followed by Tukey's post hoc test was performed for statistical analysis (error bar: ±SD; n=3; *p<0.05, **p<0.01, ***p<0.001, ****p<0.0001).
